# MicroRNA–Mediated Repression of the Seed Maturation Program during Vegetative Development in *Arabidopsis*

**DOI:** 10.1371/journal.pgen.1003091

**Published:** 2012-11-29

**Authors:** Xurong Tang, Shaomin Bian, Mingjuan Tang, Qing Lu, Shengben Li, Xigang Liu, Gang Tian, Vi Nguyen, Edward W. T. Tsang, Aiming Wang, Steven J. Rothstein, Xuemei Chen, Yuhai Cui

**Affiliations:** 1 Agriculture and Agri-Food Canada, Southern Crop Protection and Food Research Centre, London, Ontario, Canada; 2 Plant Biotechnology Institute, National Research Council of Canada, Saskatoon, Saskatchewan, Canada; 3 Department of Molecular and Cellular Biology, University of Guelph, Guelph, Ontario, Canada; 4 Department of Biology, Western University, London, Ontario, Canada; 5 Department of Botany and Plant Sciences, Center for Plant Cell Biology, Institute of Integrative Genome Biology, University of California Riverside, Riverside, California, United States of America; 6 Howard Hughes Medical Institute, University of California Riverside, Riverside, California, United States of America; Stanford University School of Medicine, United States of America

## Abstract

The seed maturation program only occurs during late embryogenesis, and repression of the program is pivotal for seedling development. However, the mechanism through which this repression is achieved in vegetative tissues is poorly understood. Here we report a microRNA (miRNA)–mediated repression mechanism operating in leaves. To understand the repression of the embryonic program in seedlings, we have conducted a genetic screen using a seed maturation gene reporter transgenic line in Arabidopsis (*Arabidopsis thaliana*) for the isolation of mutants that ectopically express seed maturation genes in leaves. One of the mutants identified from the screen is a weak allele of *ARGONAUTE1* (*AGO1*) that encodes an effector protein for small RNAs. We first show that it is the defect in the accumulation of miRNAs rather than other small RNAs that causes the ectopic seed gene expression in *ago1*. We then demonstrate that overexpression of miR166 suppresses the derepression of the seed gene reporter in *ago1* and that, conversely, the specific loss of miR166 causes ectopic expression of seed maturation genes. Further, we show that ectopic expression of miR166 targets, type III homeodomain-leucine zipper (HD-ZIPIII) genes *PHABULOSA* (*PHB*) and *PHAVOLUTA* (*PHV*), is sufficient to activate seed maturation genes in vegetative tissues. Lastly, we show that PHB binds the promoter of *LEAFY COTYLEDON2* (*LEC2*), which encodes a master regulator of seed maturation. Therefore, this study establishes a core module composed of a miRNA, its target genes (*PHB* and *PHV*), and the direct target of PHB (*LEC2*) as an underlying mechanism that keeps the seed maturation program off during vegetative development.

## Author Summary

Seed development can be conceptually divided into two phases: namely the morphogenesis phase, in which cell division is active and all the major organs are formed, and the maturation phase, in which cells enlarge and storage reserves are synthesized and accumulated. Expression of the seed maturation program is tightly controlled such that it only occurs during the late phase of seed development. To uncover the molecular mechanisms underlying the repression of seed genes during vegetative development, we performed a reporter-assisted genetic screen, and one mutant identified is a weak allele of *ARGONAUTE1* (*AGO1*) that displays ectopic seed gene expression. We then performed a series of transgenic and genetic analyses to search for the molecular mechanisms underlying the mutant phenotype. We first demonstrate that the decrease in miR166 in *ago1* is a major cause of the mutant phenotype. Further, we show that the targets of miR166, type III HD-ZIP transcription factors PHB and PHV, are sufficient for derepressing seed maturation genes in seedlings, likely by binding directly to the promoter of a master regulator gene of maturation. Thus, this work establishes a miRNA–mediated pathway that represses the embryonic program and also establishes PHB/PHV as direct activators of the maturation program.

## Introduction

Seed maturation is a highly coordinated developmental phase in which storage reserves, including seed storage proteins (SSPs), are synthesized and accumulated to high levels. The maturation genes need to be repressed, however, in order to allow seedling development to occur. Indeed, these genes are not expressed in vegetative organs of the plant [1]. Research in the past decade with the model plant Arabidopsis has led to the identification of repressors of seed maturation genes in vegetative organs (reviewed in [2]), including chromatin-remodelling ATPases [3]–[5], polycomb group (PcG) proteins [6]–[9], histone deacetylases [10], and DNA-binding transcription factors [11]–[13]. However, our understanding of the molecular mechanisms that repress the seed maturation program during vegetative development remains fragmented, and thus continued efforts are needed to identify additional factors involved and, more importantly, the molecular and functional links between the various components.

In Arabidopsis, *ABA-INSENSITIVE3* (*ABI3*), *FUSCA3* (*FUS3*), *LEC1* and *LEC2* are master regulators of seed maturation [14]–[17], and they regulate one another [18], [19]. ABI3, FUS3 and LEC2 are closely-related members of a plant-specific B3-domain transcription factor family. *LEC1* encodes a novel homolog of the CCAAT-binding factor HAP3 subunit. Loss-of-function mutations in *ABI3*, *FUS3*, and *LEC1* give rise to pleiotropic seed phenotypes including a strong reduction of SSPs. These regulatory genes are predominantly expressed in the seed. When misexpressed in vegetative tissues, they induce ectopic expression of the SSP genes and even the formation of somatic embryos [15], [17], [20]–[22]. It remains poorly understood, however, how the expression and activity of these master regulators are in turn regulated.

Small RNAs of 20–30 nucleotides (nt) have emerged as key sequence-specific regulators of gene expression that influence almost all aspects of plant biology (reviewed in [23]–[26]). There are two major types of small RNAs in plants, microRNA (miRNA) and small interfering RNA (siRNA). Plant miRNAs are generated from longer precursors arising from defined genomic loci – the *MIRNA* genes. The biogenesis of miRNAs involves several evolutionarily conserved families of proteins, including DICER-LIKE (DCL), ARGONAUTE (AGO), HUA ENHANCER 1 (HEN1), and HASTY (HST). Plant miRNAs regulate target mRNAs temporally and spatially through transcript cleavage and/or translational inhibition. Conserved miRNAs tend to target transcription factor genes that play crucial roles in almost all aspects of plant development. Plants are rich in endogenous siRNAs, which can be classified into several types, such as trans-acting siRNAs (ta-siRNAs), natural cis-antisense transcripts-associated siRNAs, and heterochromatic siRNAs.

Here, we show that mutations in *AGO1* resulted in the ectopic expression of seed maturation genes in seedlings. Taking advantage of the weak *ago1* allele identified in this work, we were able to identify the miRNA species (miR166) responsible for the repression of seed genes. We demonstrated that targets of miR166, the class III homeodomain leucine zipper (HD-ZIPIII) family of transcription factor genes, *PHB* and *PHV*, are positive regulators of seed genes. Further, we provided evidence to suggest that PHB acts directly at *LEC2*. This work thus uncovered an important role of miR166 in the repression of seed genes during seedling development.

## Results

### Identification of a Weak *ago1* Allele That Causes Ectopic Expression of a Seed Gene Promoter Reporter

**Figure 1:**
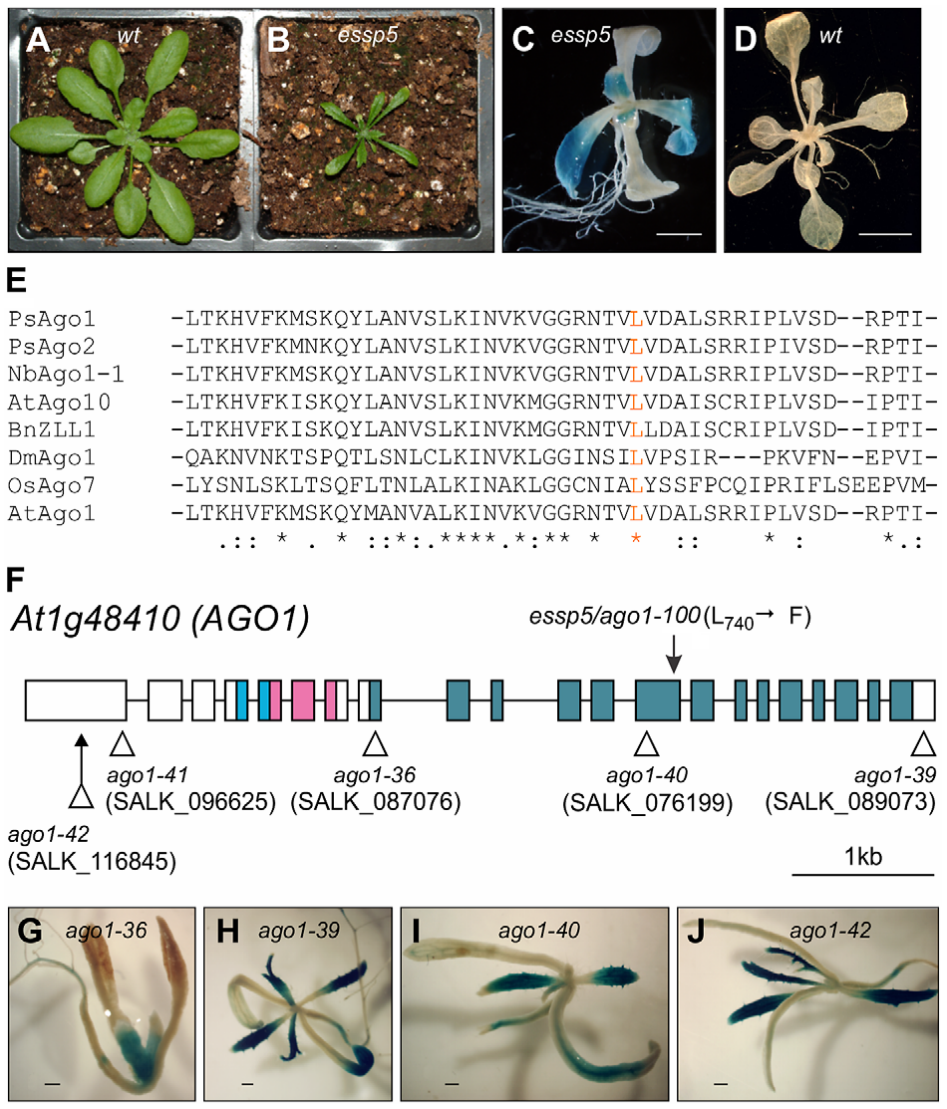
*essp5* Results in Ectopic Expression of a Seed Marker Gene and Is Allelic to *ARGONAUTE1*. (A and B) Comparison of *essp5* with wild type (wt) plants at two weeks. (C and D) GUS expression of the *essp5* mutant (C) and wild type (*wt*, *βCG:GUS*) grown on agar at two weeks. Scale bars, 2 mm in (C) and 5 mm in (D). (E) Alignment of partial amino acid sequences (residues 707 to 755) of PIWI domains in AGO proteins from Arabidopsis (At), Rice (Os), Pea (Ps), Tobacco (Nb), Drosophila (Dm), Brassica (Bn). The *essp5* mutation site (L740) is in orange. The asterisks indicate absolutely conserved residues, colons indicate high similarity, and dots indicate low similarity. (F) Structure of the *AGO1* gene showing the location of the *essp5* mutation and the T-DNA insertion sites of other *ago1* alleles. Boxes and lines represent exons and introns, respectively. The colored boxes represent the conserved protein domains: light blue (PAZ), magenta (MID), and blue (PIWI). (G–J) GUS expression in four T-DNA insertion alleles of *AGO1*. Shown here is a representative F_2_ progeny from each of the crosses of the corresponding T-DNA allele with the *βCG:GUS* line. Scale bar, 500 µm.

We have recently conducted a genetic screen in *Arabidopsis* to isolate mutants exhibiting ectopic expression of a soybean β-conglycinin gene promoter:GUS transgene (*βCG:GUS*), which is normally expressed only in the seed [5], [27], [28]. Here, we describe the characterization of one of the mutants identified from the screen, initially named *essp5* (*ectopic expression of seed storage proteins 5*). The *essp5* mutant plants exhibited strong ectopic GUS activity in leaves, but not in other organs (Figure 1A–1D). In addition, the mutant plants had pleiotropic developmental defects, such as late flowering, narrow and dark green leaves, shorter siliques and fewer seeds (Figure 1B and Figure S1).

The mutation segregated as a recessive allele and was mapped to the *AT1G48410* gene (Figure S2), which encodes AGO1, the major effector protein that associates with small RNAs [29]. A single missense mutation in this gene was identified that would lead to the conversion of a leucine residue at position 740 to a phenylalanine. The leucine 740 is a highly conserved residue in the PIWI domains of AGOs from diverse species (Figure 1E). A number of mutant *ago1* alleles have been described previously and their pleiotropic morphological phenotypes have been documented [29]–[32]. The morphological phenotypes of *essp5* resemble those documented for weak *ago1* alleles. We obtained T-DNA insertion mutants of *ago1*, including *ago1-36* (SALK_087076), *ago1-39* (SALK_089073), *ago1-40* (SALK_076199), *ago1-41* (SALK_096625), and *ago1-42* (SALK_116845) (Figure 1F), crossed them with *βCG:GUS* and examined GUS expression in F2 progeny seedlings. The T-DNA insertion lines, regardless of their insertion sites, all displayed similar morphological phenotypes: long rod-shape cotyledons, delayed emergence of true leaves, and premature death with only a couple of small true leaves. As shown in Figure 1G–1J, ectopic GUS activity was clearly observed in several T-DNA alleles with insertion sites located throughout the gene. Furthermore, we performed an allelism test to provide additional evidence that *essp5* is a weak *ago1* allele. A weak *ago1* allele (*ago1-25*) that exhibits similar morphological phenotype [33] was crossed with *essp5* and the F1 progeny were examined for GUS activity. As shown in Figure S3, the F1 seedlings displayed ectopic GUS expression, indicating that the *essp5* GUS phenotype cannot be complemented by a weak *ago1* allele. Together, these observations suggest that *essp5* is allelic to *AGO1* and thus designated as *ago1-100*.

### Ectopic Expression of Seed Genes in *ago1* Mutant Seedlings

**Figure 2:**
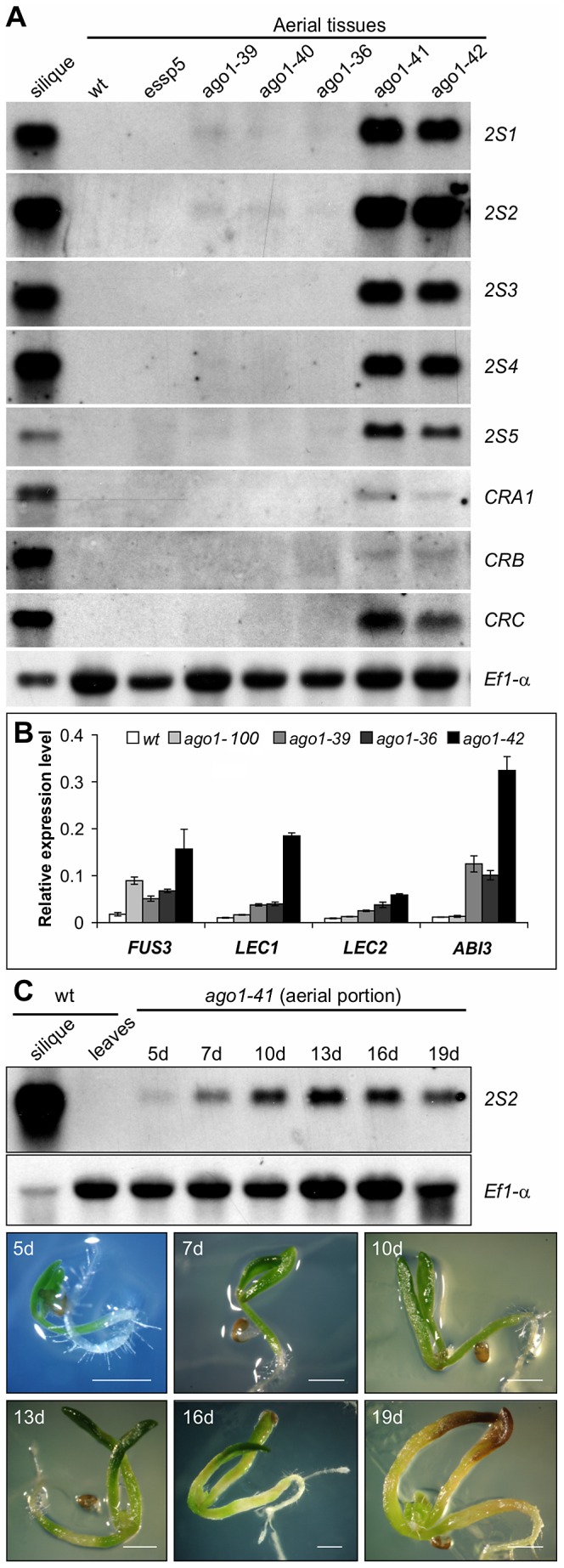
Expression Analysis of Seed Maturation Genes in *ago1* Mutant Seedlings. (A) RNA blot analysis of the expression of the five *2S* genes and three 12S genes (*CRA1*, *CRB*, and *CRC*) in *ago1* mutant seedlings grown for 14 days on MS agar. Wild type (*Col*) leaves and siliques were used as negative and positive controls, respectively. Same amount of RNA was used for each blot. Elongation factor 1α was used as loading control. (B) Real time quantitative RT-PCR (qRT-PCR) analysis of *ABI3*, *FUS3*, *LEC1*, and *LEC2* genes in seedlings (aerial portion) of four *ago1* mutants grown for 12 days on MS agar. *Actin-8* was used as an internal control. The mean and standard error were determined from three biological replicates, for each of which the PCR was conducted in triplicated technical repeats. (C) RNA blot analysis of temporal expression of maturation genes (*2S2* as a marker) in *ago1-41* mutant seedlings grown on MS agar for 5, 7, 10, 13, 16, and 19 days. Wild type (*Col*) leaves and siliques were used as negative and positive controls, respectively. Same amount of RNA was used for each blot. Elongation factor 1α was used as a loading control. Shown at the bottom are images of the *ago1-41* mutant on agar at various time points. Scale bar, 1 mm.

To find out whether the endogenous seed maturation genes are indeed ectopically expressed in *ago1* mutant seedlings, we performed northern blot analysis to profile the expression of both the *2S* and *12S* storage protein genes. As shown in Figure 2A, the transcripts of the storage protein genes are highly accumulated in the two strong alleles, *ago1-41* and *ago1-42*, both with T-DNA insertion sites located in the 5′ end of the gene but barely detectable in *ago1-100*/*essp5* and other weak alleles with insertion sites located in the middle and 3′ end of the *AGO1* gene. We further examined the transcript levels of the four master regulators by quantitative real-time RT-PCR (qRT-PCR). In line with the ectopic expression of the storage protein genes, all the master regulators are expressed to varying levels in the mutants, especially *ago1-42* (Figure 2B). In addition, we also profiled the temporal expression pattern of the maturation genes, using *2S2* as a marker. *ago1-41* seedlings from 5 d to 19 d after germination were examined. As shown in Figure 2C, the *2S2* transcript peaked in abundance at around 13 d, but was clearly detected throughout the time course. These expression analyses clearly demonstrate the involvement of AGO1 in the repression of seed maturation genes.

### Derepression of Seed Maturation Genes in Other miRNA Biogenesis Mutants

**Figure 3:**
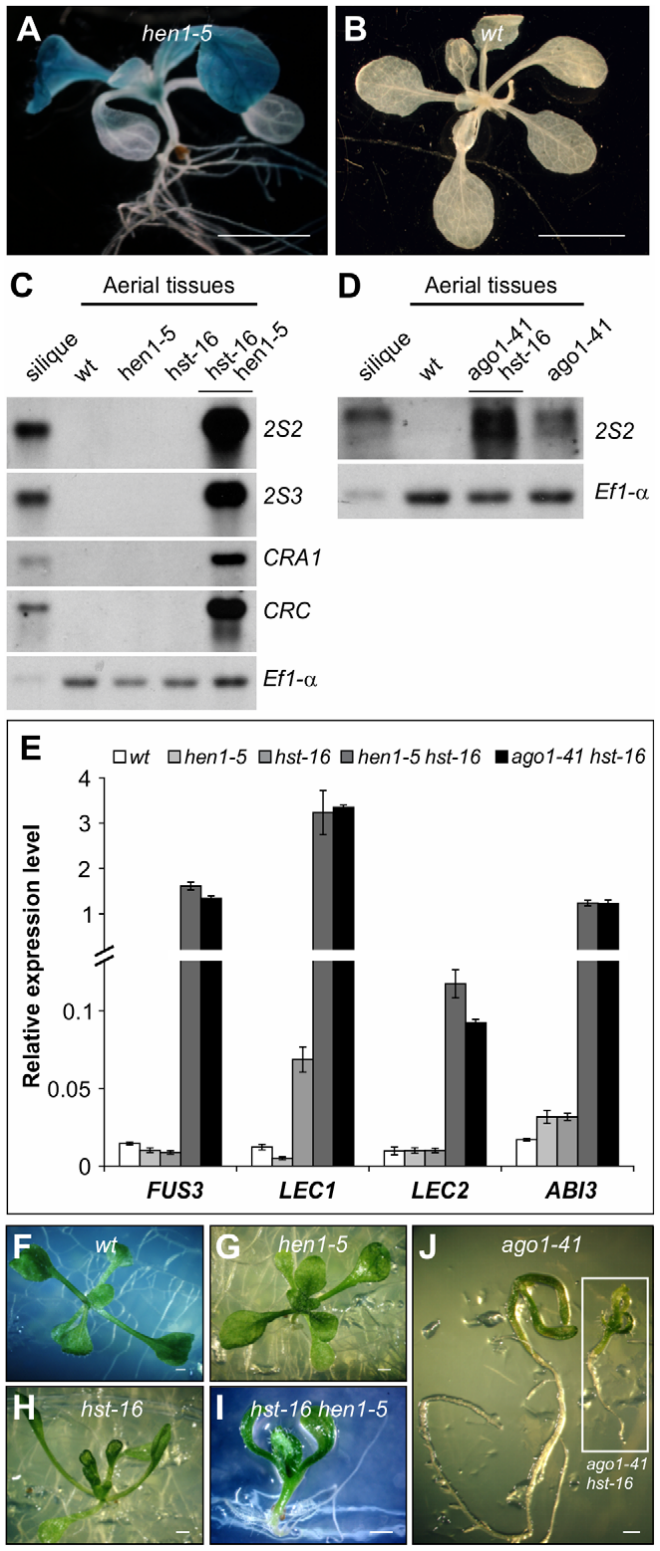
Ectopic Expression of Seed Maturation Genes in miRNA Biogenesis Mutants. (A and B) GUS expression in *hen1* mutant (A) and wild type (*wt, βCG:GUS*) grown on agar for two weeks. Shown here are representative F_2_ progeny from the crosses of *hen1-5* with the *βCG:GUS* line. Scale bars, 3 mm in (A) and 5 mm in (B). (C and D) RNA blot analysis of the *2S* and *12S* genes in seedlings (aerial tissues) in various miRNA biogenesis mutant backgrounds grown for 14 days on MS agar. Wild type (*Col*) leaves and siliques were used as negative and positive controls, respectively. Same amount of RNA was used for each blot. Elongation factor 1α was used as a loading control. (E) qRT-PCR analysis of *ABI3*, *FUS3*, *LEC1*, and *LEC2* genes in seedlings (aerial portion) of various miRNA biogenesis mutants grown for 15 days on MS agar. *Actin-8* was used as an internal control. The mean and standard error were determined from three biological replicates. (F–J) Morphological phenotypes of 13-day seedlings of various miRNA biogenesis mutants. Scale bar, 1 mm.

AGO1 associates with miRNAs and some endogenous siRNAs to mediate their activities [34]. AGO1 association also stabilizes the small RNAs such that *ago1* mutants show a reduction in the steady-state levels of miRNAs and siRNAs [35]–[36]. To confirm that the ectopic expression of seed maturation genes in *ago1* mutants is due to defects in small RNA biogenesis or activity, we examined seed gene expression in seedlings of loss-of-function alleles of genes commonly involved in small RNA biogenesis. For this purpose, we obtained mutant alleles of *HEN1*, *hen1-5* (SALK_049197) and *hen1-6* (SALK_090960), and of *HST*, *hst-1* [37], *hst-15* (SALK_079290) and *hst-16* (SALK_056352). We introduced these mutations, individually, into the *βCG:GUS* background and examined GUS expression in the F2 generation. We were able to detect clear ectopic GUS activity in the *hen1* backgrounds (*hen1-5*; Figure 3A and 3B), but not in the *hst* alleles. We then generated double mutants between *ago1*, *hen1*, and *hst*. As shown in Figure 3C–3J, both *ago1-41 hst-16* and *hen1-5 hst-16* double mutants exhibited a high level of expression of the storage protein genes and the master regulators. The *ago1-41 hen1-5* plants were very small, which precluded seed gene expression analysis. These results indicate synergistic genetic interactions among *AGO1*, *HEN1*, and *HST* in repressing seed genes during seedling development and, more importantly, the involvement of a small RNA pathway(s) in this repression process.

Since AGO1, HEN1 and HST are essential players in small RNA biogenesis and are involved in several small RNA pathways [38], it was necessary to determine which pathway underlies the mutant phenotype. To this end, we took advantage of pathway-specific components to define the specific pathway responsible for the *ago1* mutant phenotype. Specifically, RDR2 is an essential component of the heterochromatin pathway and RDR6 is required for the biogenesis of trans-acting siRNAs. We obtained and introduced the *rdr2-1* (SAIL_1277_H08) and *rdr6-11* [39] mutations into the *βCG:GUS* background by genetic crosses and examined GUS expression in the F2 progeny. A large number of F2 seedlings were stained for GUS and no ectopic GUS activity was observed in either population. This genetic evidence suggests that it is unlikely that the trans-acting siRNA or the hc-siRNA pathway is involved in the repression of seed genes in seedlings. Since AGO1, HEN1, and HST all act in miRNA biogenesis, a miRNA(s) is thought to be a strong candidate for the repression of seed genes during vegetative development.

### Reduced Accumulation of Conserved miRNAs in *ago1* and Other miRNA Biogenesis Mutants

**Figure 4:**
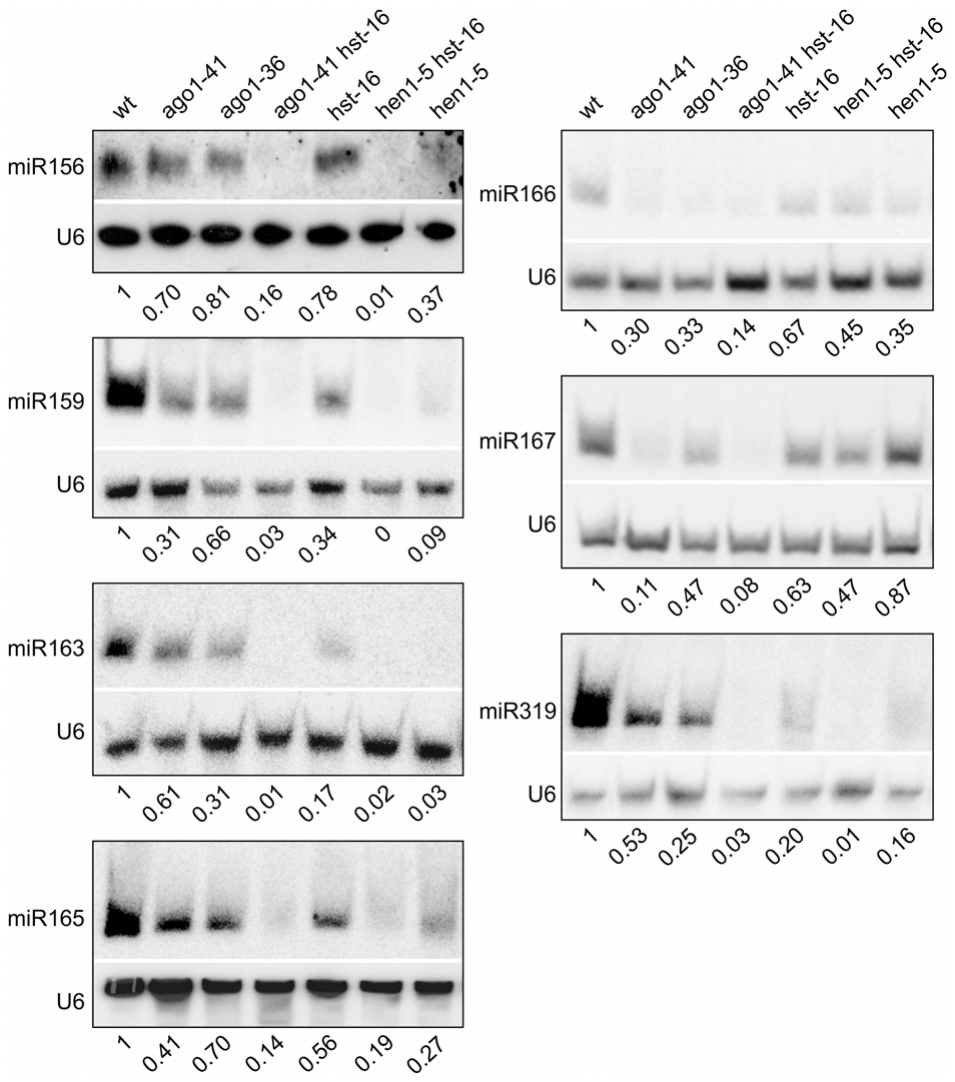
Reduced Accumulation of miRNAs in Various miRNA Biogenesis Mutant Backgrounds as Determined by Northern Blotting. The genotypes of the mutants are indicated at the top. Total RNAs were extracted from two-week old seedlings (aerial portion). The levels of each small RNA were normalized to those of U6 and compared with wild type (Col). The numbers below the gel images indicate the relative abundance of the small RNAs.

To provide evidence that a miRNA pathway is indeed underlying the mutant phenotype, we examined the steady-state levels of a number of conserved miRNAs in *ago1* and other mutant backgrounds. In *ago1* mutants, it was documented previously that the accumulations of a number of conserved miRNAs decline markedly and their target gene transcripts are concomitantly elevated [35]–[36]. Here, we performed a miRNA northern blot analysis to examine and compare the accumulation of conserved miRNA species in *ago1*, *hen1*, *hst*, and the two double mutants, *ago1 hst* and *hen1 hst*. As shown in Figure 4, we confirmed the published observation for *ago1* in that all the miRNAs examined were clearly reduced. More importantly, we observed further reduced accumulation of most examined miRNAs in *ago1 hst* and *hen1 hst* double mutants compared to the single mutants (Figure 4). These findings are consistent with a genetic model for explaining the ectopic seed gene expression in *ago1*and other mutants: the steady-state level of a specific miRNA was reduced below a threshold to lead to the ectopic expression of its target gene, which encodes a positive regulator of seed maturation genes leading to the ectopic expression of seed genes in leaves.

### Overexpression of miRNA166 Rescues the *essp5*/*ago1-100* Mutant Phenotype and Loss of miR166 Causes Ectopic Expression of Seed Maturation Genes

Post-germination repression of seed genes is critical in order for the seedling to develop normally. We thus reasoned that such a fundamental developmental program should be controlled by a conserved miRNA(s). Therefore, to find out which miRNA was involved in conferring the *essp5*/*ago1-100* GUS phenotype, we over-expressed each of the 15 conserved miRNA species, as listed in [23] and Table S1, in the *essp5*/*ago1-100* background and examined GUS expression of the resulting transgenic plants. The transgenic plants overexpressing different miRNAs displayed unique morphological phenotypes, which are consistent with previously published observations (reviewed in [23]).

**Figure 5:**
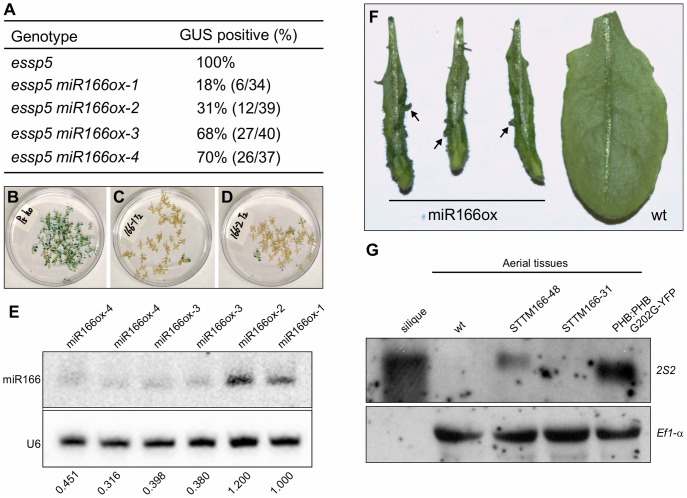
Over-Expression of miRNA166 Rescues the *essp5* GUS Phenotype, and Loss of miR166 Causes Ectopic Expression of Seed Maturation Genes. (A) Loss of ectopic GUS activity in *essp5* plants overexpressing miR166. The miR166 transgene driven by the CaMV 35S promoter was introduced into *essp5/+* background. Four T1 transgenic lines were identified, designated *essp5 miR166ox-1–4*. T2 seeds were germinated on selective MS agar plates such that only seedlings transgenic for miR166 were selected. After 13 days of growth, *essp5*-like seedlings were harvested and stained for GUS activity. (B–D) Photos showing the GUS phenotypes of *essp5* (B), *essp5 miR166ox-1* (C), and *essp5 miR166ox-2* (D), respectively, as listed in (A). (E) Accumulation of miR166 in the four transgenic lines as listed in (A), i.e., *essp5 miR166ox-1–4*, as determined by northern blotting. The genotypes of the plants are indicated at the top. Total RNAs were extracted from two-week old seedlings (aerial portion). The numbers below the gel images indicate the relative abundance of the small RNAs. (F) Comparison of leaf morphology of miRNA166 overexpressors with that of wild type. Note the aberrant structures formed on the transgenic leaves (arrows). (G) RNA blot analysis of the *2S2* gene in seedlings (aerial tissues) in the miR166 silencing lines STTM165/166-48 and STTM165/166-31 grown for 14 days on MS agar. Wild type (*Col*) leaves and siliques were used as negative and positive controls, respectively. The *PHB:PHB G202G-YFP* transgenic plants were also included as a positive control. Elongation factor 1α was used as a loading control.

Analysis of leaf GUS expression was conducted in the T2 generation. For each miRNA transgene, multiple independent transgenic lines were analyzed (in most cases 10 lines); and for each line, at least 20 T2 progeny homozygous for *essp5* were stained for GUS activity. We only observed loss of leaf GUS activity in miR166 and miR156 overexpressing lines. In this study, we have focused on the characterization of miR166. In total, we only obtained four miR166 transgenic lines, *miR166ox-1-4*, of which two showed clear loss of leaf GUS activity (*miR166ox-1-2*) while the other two (*miR166ox-3-4*) did not show as obvious a change compared with *essp5*/*ago1-100* seedlings (Figure 5A–5D). The extremely low rate of positive transgenic plants for miR166 is likely due to the fact that some transgenic seedlings failed to develop the shoot apical meristem and could not survive in soil, as observed by others [40]. To confirm that the loss of leaf GUS activity in the transgenic lines was indeed due to the elevated accumulation of miR166, a northern blot analysis was performed. As shown in Figure 5E, there were clearly higher levels of miR166 in lines *miR166ox-1-2* than lines *miR166ox-3-4*. In addition, we observed the formation of aberrant structures on leaves of *miRNA166ox-1-2* (Figure 5F). Similar aberrant structures were observed by Zhou et al in miRNA166 overexpressors [40]. These observations suggest that the reduction of miRNA166 and the concomitant accumulation of its target gene transcripts are likely the cause underlying the ectopic GUS phenotype of *essp5*/*ago1-100* seedlings.

To demonstrate that the specific loss of miR166 can cause the ectopic expression of seed maturation genes, we obtained the recently developed transgenic lines that exhibit a dramatic reduction in miR165/166 accumulation achieved by the expression of a short tandem target mimic (STTM165/166) [41]. RNA blot analysis was performed to examine the expression of seed storage protein genes in these transgenic lines, using *2S2* as a probe. As shown in Figure 5G, the *2S2* gene is clearly expressed in the strongest line (STTM165/166-48), but not detectable in a weaker line (STTM165/166-31). This observation indicates that miR166 plays an important role in repressing seed genes in seedlings.

### Ectopic Expression of the HD-ZIPIII Genes *PHB* or *PHV* Is Sufficient for Seed Gene Expression in Seedlings

It has been well established that the miR165/166 family miRNAs target the transcripts of the HD-ZIPIII genes, controlling their expression level and domain, to fulfill their roles in plant development including leaf polarity determination [42]–[45]. The HD-ZIPIII family consists of five transcription factors (REV, PHB, PHV, AtHB8, and AtHB15), and they play both redundant and unique roles in diverse plant developmental processes [46]. In this context, it is worth noting that the transcript level of *PHB* was found to be decreased in miR166 overexpressors (Figure S4). To investigate whether the HD-ZIPIII proteins are responsible for conferring the ectopic GUS phenotype of *essp5*/*ago1-100*, we introduced loss-of-function mutations in *PHB* and *PHV* genes into *essp5*/*ago1-100* by genetic crosses and examined leaf GUS expression in F2 and F3 seedlings. A large number of F2/F3 seedlings were examined and no clear loss of leaf GUS activity was observed in *phb essp5* or *phv essp5*. We further introduced *phb phv* double mutations into the *essp5*/*ago1-100* background, but still saw no detectable loss of leaf GUS activity. Obviously, the potential redundancy among the five HD-ZIPIII genes could be confounding the genetic analyses above.

**Figure 6:**
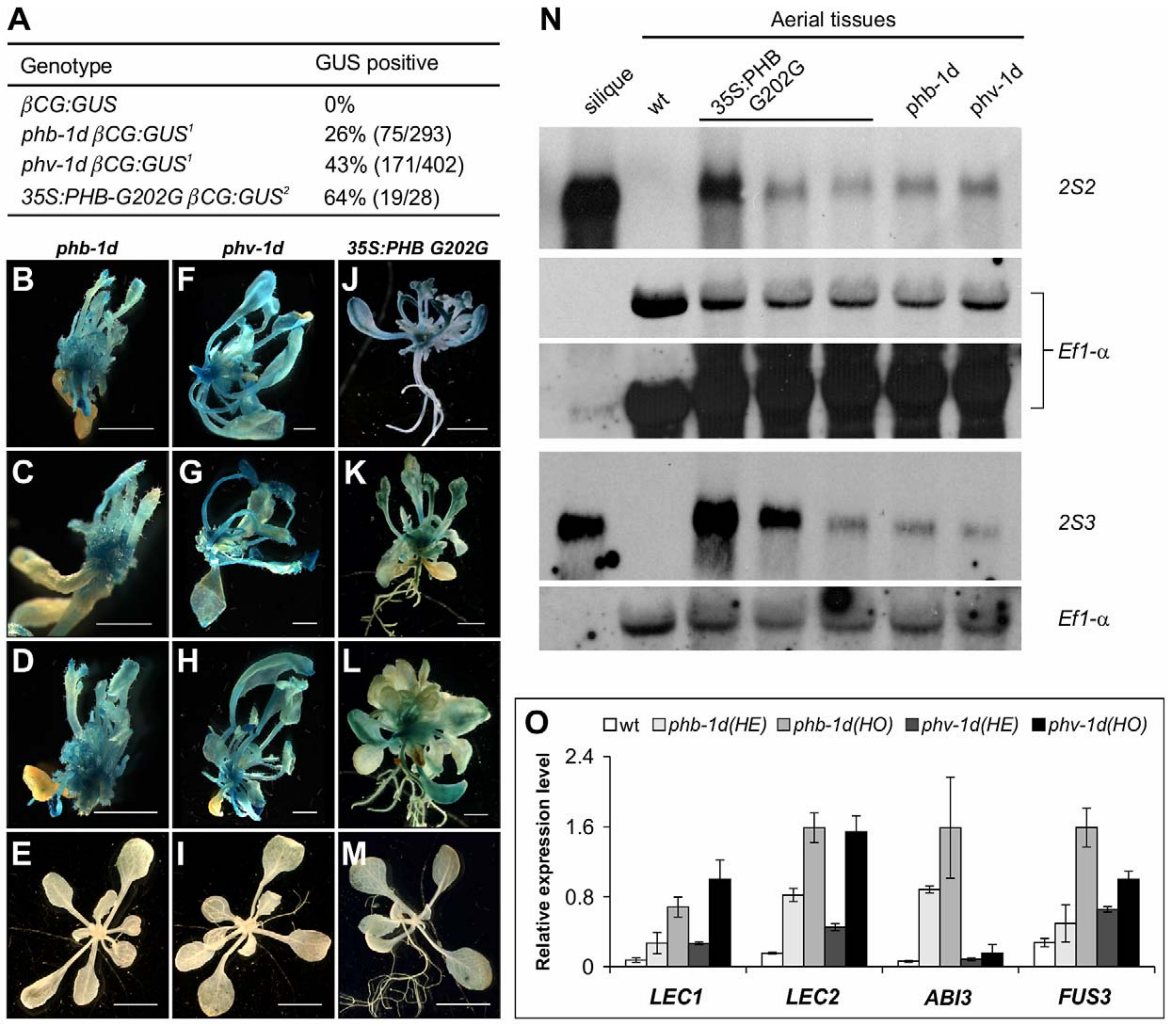
HD-ZIPIII Genes *PHB/PHV* Are Sufficient for Activating Ectopic Expression of Seed Genes in Seedlings. (A) GUS phenotypes of gain-of-function mutations in *PHB* and *PHV* genes. Note^1^: *phb-1d* or *phv-1d* was crossed with *βCG:GUS*. In the F2 progeny grown on agar for three weeks, *phb-1d- or phv-1d*-like plants were selected and stained for GUS activity. Note^2^: The *35S:PHB G202G* transgene was introduced into the *βCG:GUS* background by agrobacterium-mediated transformation. Independent T1 transgenic seedlings were selected and stained for GUS activity. (B–M) GUS phenotypes of gain-of-function *phb* and *phv* mutants grown on agar for three weeks. Shown here are representative F_2_ progeny from the cross of *phb-1d* or *phv-1d* with *βCG:GUS*, as well as the T1 transgenic lines containing the *35S:PHB G202G* in *βCG:GUS* background. Wild type (*wt*, *βCG:GUS*) seedlings (two weeks old) were also stained as negative controls (E, I, and M). Scale bars, 2 mm in B–D, F–H and J–K; 5 mm in E, I and M. (N) RNA blot analysis of the expression of the maturation genes (*2S2* and *2S3* as markers) in the gain-of-function *phb* and *phv* mutant seedlings grown for 22 days on MS agar. For the *35S:PHB G202G* transgenic plants, independent T1 plants were selected and grouped into three groups according to the severity of their morphological phenotypes. Note the difference in the hybridization intensities. Wild type (*Col*) leaves and siliques were used as negative and positive controls, respectively. Same amount of RNA was used for each blot. Elongation factor 1α was used as a loading control. In the top panel, two exposures of the *EF1- α* loading control were shown. (O) qRT-PCR analysis of *ABI3*, *FUS3*, *LEC1*, and *LEC2* expression in seedlings (aerial portion) of *phb-1d* and *phv-1d* mutants grown for 20 days on MS agar. For each mutant allele, heterozygous (HE) and homozygous (HO) mutant seedlings were measured separately for comparison. *Actin-8* was used as an internal control. The mean and standard error were determined from three biological replicates.

Next, taking advantage of the previously identified gain-of-function mutations in HD-ZIPIII family genes, we investigated whether these proteins are sufficient to cause the ectopic expression of seed genes. These gain-of-function alleles have mutations in the miR166 target regions to cause a mismatch between the miRNA and the target mRNA and thus render the transcripts resistant to miRNA-mediated degradation and consequently the ectopic accumulation of HD-ZIPIII transcripts. First, the gain-of-function mutations *phb-1d* [47] and *phv-1d* [44] were introduced into the *βCG:GUS* background by genetic crosses and ectopic GUS activity was examined in F2 seedlings (Figure 6A–6I). Meanwhile, another gain-of-function *phb* allele driven by the CaMV 35S promoter, *35S:PHB G202G* [43], was also introduced into the *βCG:GUS* background by *Agrobacterium*-mediated transformation and GUS activity was examined for each independent T1 plant (Figure 6A and 6J–6M). As shown in Figure 6A–6M, ectopic GUS activity was clearly observed for *phb-1d*, *phv-1d*, and *35S:PHB G202G*. Further, we performed northern blot and qRT-PCR analyses to examine the ectopic expression of endogenous seed maturation genes in the gain-of-function mutant plants (Figure 6N and 6O). Two representative maturation genes *2S2* and *2S3* were clearly expressed in the mutant seedlings (Figure 6N). Similarly, the four master regulators were all elevated to varying levels (Figure 6O). In addition, a gain-of-function *REV* mutant, *rev-10d* [42], was also analyzed in the *βCG:GUS* background but no ectopic GUS activity was detected. In summary, our gain-of-function genetic evidence indicates that the HD-ZIPIII proteins PHB and PHV are each sufficient for ectopic expression of seed genes.

### Physical Occupancy of PHB at the *LEC2* Gene Promoter

HD-ZIP proteins are plant-specific transcription factors and named for the combination of homeodomain and leucine zipper domains at their N termini [48]. They bind a palindromic DNA sequence *in vitro* as dimers [49]. To determine whether PHB acts directly at maturation gene loci, we performed chromatin immunoprecipation (ChIP) experiments to examine PHB occupancy at the promoters of these genes.

**Figure 7:**
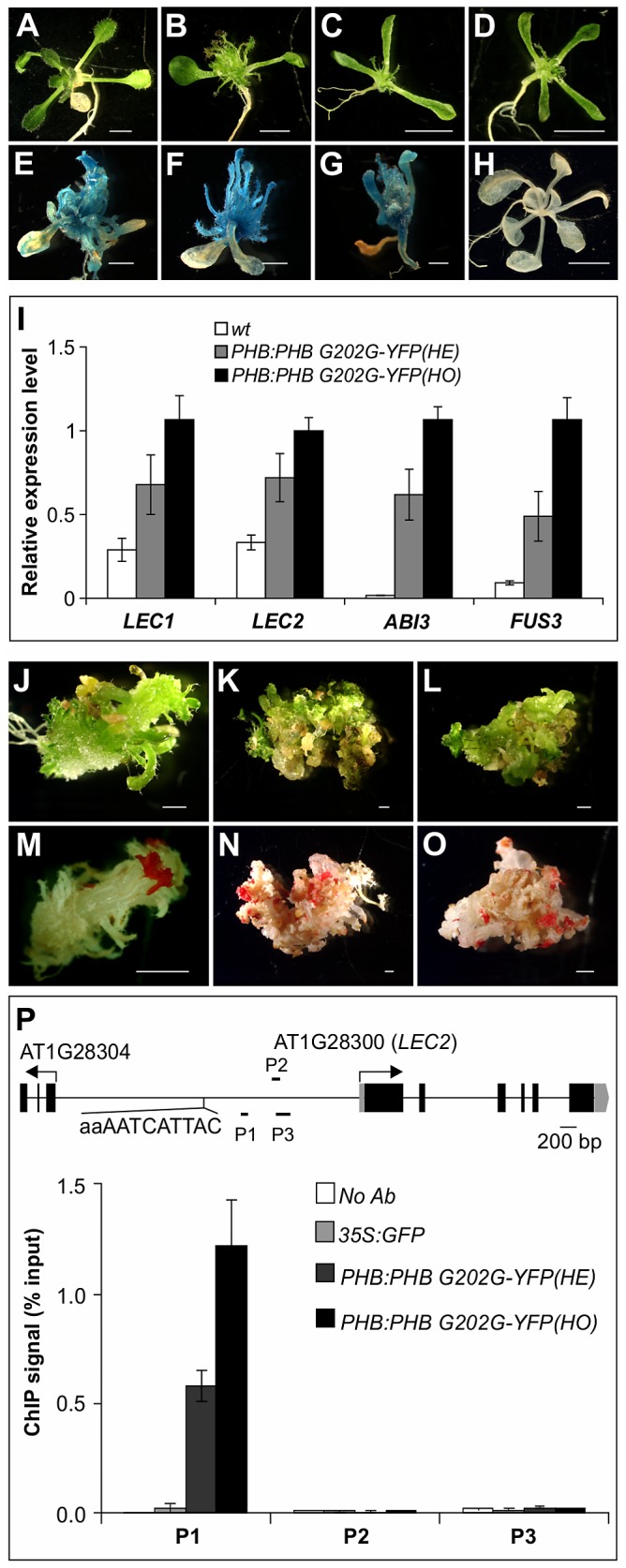
PHB Occupancy at *LEC2* Promoter. (A–D) Morphological phenotype of the *35S:GFP* (A) and *PHB:PHB G202G-YFP* (B–D) transgenic plants grown on agar for three weeks. (B) a hemizygous plant. (C–D) homozygous plants. Note that multiple cotyledons (3 or 4) were found in the majority (∼80%) of *PHB:PHB G202G-YFP* homozygous plants. Scale bar, 3 mm. (E–H) GUS phenotype of *PHB:PHB G202G-YFP* transgenic seedlings (in *βCG:GUS* background) grown on MS agar for three weeks (E–G) and wild type (*wt*, *βCG:GUS*) grown on MS agar for two weeks. Scale bars, 1 mm in E–G and 5 mm in H. (I). qRT-PCR analysis of *ABI3*, *FUS3*, *LEC1*, and *LEC2* expression in seedlings (aerial portion) of hemizygous (HE) and homozygous (HO) *PHB:PHB G202G-YFP* seedlings grown for 20 days on MS agar. *Actin-8* was used as an internal control. The mean and standard error were determined from three biological replicates. (J–O) Fat red staining of *PHB:PHB G202G-YFP* plants that exhibited disorganized growth. Such plants were found at a rate of approximately 10% among the homozygous transgenic plants. Shown in J (20 d), K (35 d), and L (35 d) are plants before staining that correspond to those in (M), (N), and (O), respectively, after staining. Scale bar, 1 mm. (P) PHB occupancy at *LEC2* promoter as determined by ChIP using anti-GFP antibody in *PHB:PHB G202G-YFP* plants. The *35S:GFP* plants served as negative control. ChIP DNAs were analyzed by qPCR. The results were reproduced in two biological replicates. Standard deviations were calculated from three technical repeats. Shown at the top is a schematic representation of the *LEC2* gene. Boxes and lines represent exons and introns, respectively. Transcription start sites are indicated by arrows. Black bars labeled P1–P3 represent the regions examined by ChIP-qPCR. The potential PHB binding sequence is also indicated.

For the ChIP assays, we generated *Arabidopsis* plants transgenic for a YFP-tagged gain-of-function allele of *PHB* under the control of the *PHB* native promoter (*PHB:PHB G202G-YFP*). Morphologically, the transgenic plants resemble the *phb-1d* mutant (Figure 7A–7D). When the transgene was introduced into the *βCG:GUS* background, it resulted in ectopic GUS activity (Figure 7E–7H). Expression of the master regulators of seed maturation in the transgenic seedlings was also examined by qRT-PCR. As shown in Figure 7I, these genes were ectopically expressed to similar levels compared to those of *phb-1d*, and the expression levels in homozygous seedlings were clearly higher than those in the hemizygous siblings. These observations demonstrate that the *PHB:PHB G202G-YFP* plants resemble *phb-1d*. In addition, we observed, at a low frequency, disorganized growth and/or formation of somatic embryo-like structures in the transgenic plants (Figure 7J–7L). Some parts of these plants could be stained by the neutral lipid dye fat red (Figure 7M–7O), indicating a high level accumulation of seed storage-specific triacylglycerols in these plants (fat red/sudan red stains only seed storage-specific lipids).

**Figure 8:**
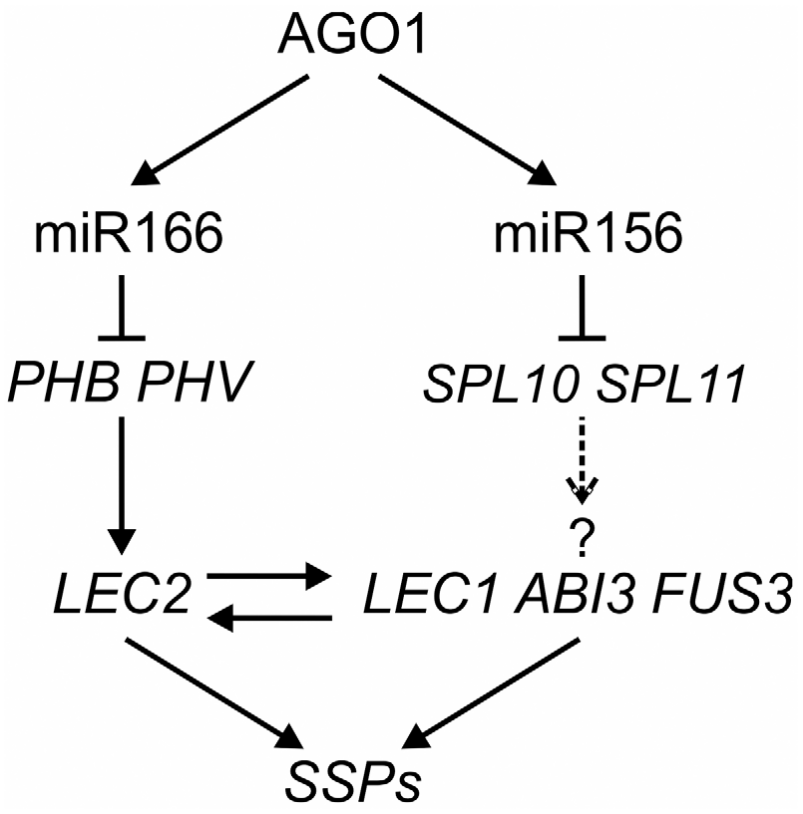
A Model of MicroRNA–Mediated Repression of Seed Genes in Leaves. In wild type seedlings, AGO1 and miR166 repress the expression of *PHB*/*PHV*, which promotes the seed maturation program by directly activating *LEC2* expression and indirectly that of other positive regulators of the seed maturation program. miR156 may also play a role in the process, possibly by repressing *SPL10* and *SPL11*, based on this work and that of Nodine and Bartel [53]. It is not known, however, whether SPLs are sufficient for inducing the expression of seed genes or whether they directly activate the seed genes.

ChIP was performed with anti-GFP antibodies and an *Arabidopsis* line transgenic for *GFP* driven by the CaMV 35S promoter (*35S:GFP*) was used as a negative control. The ChIP DNAs were analyzed by qPCR to examine the enrichment of promoter region genomic DNAs of the four master regulator genes. One region in the *LEC2* promoter was highly enriched relative to the *35S:GFP* and no antibody controls (Figure 7P), but no enrichment was found for the promoter regions of other maturation genes examined (Figure S5). The enrichment level in homozygous plants was about double that in the hemizygous siblings, consistent with the ectopic expression level of seed genes in these plants (Figure 7I). Interestingly, a partial palindromic sequence, aaAATCATTAC, was found in the vicinity of the enriched genomic region in the *LEC2* promoter, but not at other maturation loci. This sequence is very similar to the HD-ZIPIII binding consensus sequence, GTAAT(G/C)ATTAC, derived from an *in vitro* binding site selection experiment [49]. These observations suggest that PHB, when ectopically expressed, binds to the *LEC2* promoter and activates the expression of the gene. LEC2 can, in turn, activate a network of maturation-related genes including *ABI3*, *FUS3*, *LEC1*, and the SSP genes (Figure 8).

## Discussion

In this work, we first identified a weak EMS *ago1* allele, which exhibited ectopic expression of a GUS reporter driven by a seed gene promoter. Taking advantage of the weak *ago1* allele and its GUS phenotype, we then performed a series of transgenic and genetic analyses to search for the molecular mechanisms underlying the mutant phenotype. We first demonstrated that miR166 reduction is a major cause of the mutant phenotype and further showed that the targets of miR166, *PHB* and *PHV*, are sufficient for derepressing seed maturation genes in seedlings. Finally, our ChIP assay using a tagged *PHB* transgenic line suggests that PHB may act directly at the *LEC2* promoter (summarized in Figure 8). However, in addition to *LEC2*, PHB may also regulate other factors that in turn regulate seed maturation genes directly or indirectly. Future studies, such as ChIP-seq, are needed to address this issue. Therefore, this work has added miR165/166 to the documented repertoire of postgermination repressors of the embryonic program (reviewed in [2]), and more importantly, established PHB, and possibly PHV, as direct positive regulators of the master regulator of seed maturation *LEC2*. A major future challenge in the field is to find out the genetic and molecular relationships amongst the various players, including transcription factors, chromatin remodelers and modifiers, and the newly added miRNA, and build an integrated genetic network.

Given the well-established expression patterns and roles of miR166 and its targets in leaf polarity determination (reviewed in [50], [51]), an obvious outstanding question is why the normal expression of the *PHB* and *PHV* genes in the adaxial domain of leaf primordia in wild type plants is not sufficient to cause the ectopic expression of seed maturation genes. miR165/166 is concentrated in the abaxial domain to restrict the expression of the HD-ZIPIII transcription factor genes to the adaxial domain in the lateral organs in *Arabidopsis* [42]–[44] and maize [45]. In *phb* and *phv* gain-of-function mutants, the expression of *PHB* and *PHV* is not restricted to the adaxial domain but extends into the entire primordium. We observed ectopic expression of seed maturation genes only in these gain-of-function mutants, indicating that the normal, adaxial expression of the HD-ZIP III genes is not sufficient to activate the seed maturation program. There could be at least two underlying reasons. First, the ectopic expression of the seed maturation genes in the *phb* and *phv* gain-of-function mutants only occurs in the abaxial domain. In this scenario, the lack of necessary co-factors or the presence of negative factors in the adaxial domain may prevent the HD-ZIPIII genes from activating the seed maturation genes. Alternatively, it might be a matter of thresholds – the adaxial domain normally does not have sufficient levels of HD-ZIPIII expression to trigger the seed maturation program, but when the miRNA is compromised, the expression level is high enough to trigger the program. Our preliminary observation is in support of the first scenario. GUS expression along the adaxial-abaxial axis in *essp5*/*ago1-100* was examined and GUS activity was found only on the abaxial side (Figure S6). In addition, interestingly, GUS was also observed in both the upper and lower epidermal cells (Figure S6).

The seed maturation program is a tightly regulated developmental process. Mechanisms are in place to not only ensure its repression during seedling development but also prevent its precocious induction during early embryogenesis [2], [52]. The induction of seed maturation is also referred to as the morphogenesis-to-maturation phase transition of embryogenesis. While our studies have established miR165/166 and implicated miR156 as players in the repression of the seed maturation program in vegetative development, two recent studies have also revealed important roles of miRNAs in regulating the morphogenesis-to-maturation phase transition [53], [54]. Of these, the work of Nodine and Bartel [53] demonstrated that miR156 and two of its target genes *SPL10* and *SPL11* play a major role in early embryo patterning and in preventing the precocious expression of maturation genes. An obvious question is whether miR165/166 also acts similarly in early embryogenesis to control the morphogenesis-to-maturation phase transition. Previous studies have shown that *PHB* and *PHV* promote embryonic development, and that the expression of these genes must be repressed by miR165/166 for embryonic development to proceed normally. For example, Grigg et al showed that *serrate* (*se*) mutants cause ectopic expression of *PHB* and *PHV* in the root pole of embryos, and that the embryonic lethal phenotype of *se* mutants can be rescued by loss-of-function mutations in *PHB* and *PHV* [55]. Smith and Long also showed that *PHB* and *PHV* promote shoot development during embryogenesis [56]. These studies focused on the roles of the miR165/166-*PHB/PHV* module in early embryo patterning. Our finding that this module plays an important role in repressing seed maturation genes during seedling development prompted us to test its role in the morphogenesis-to-maturation phase transition. We performed a ChIP analysis using a transgenic line expressing a tagged *PHB* driven by its endogenous promoter (*PHB:PHB-YFP*). Preliminary data suggests that PHB acts directly at *LEC2* during embryogenesis (Figure S7). Future investigations are needed to sort out the contributions of each miRNA to the repression of the seed maturation program during the pre- and post-maturation stages.

## Materials and Methods

### Plant Material, Growth Conditions, and Genotype Analysis

Seeds of mutants including the T-DNA insertion mutants were obtained from the ABRC, unless otherwise indicated. Seeds were stratified at 4°C for 3-d. Then the seeds were sowed on soil or on agar plates containing 4.3 g/L Murashige and Skoog nutrient mix (Sigma-Aldrich), 1.5% sucrose, 0.5 g/L MES (pH 5.7), and 0.8% agar. Plants were grown under 16 h-light (22°C)/8 h-dark (20°C) cycles; except that the *phb-1d/+* and *phv-1d/+* mutants were grown at 17°C during reproductive development as described [47]. Homozygous T-DNA insertion mutants were identified by genotyping.

### Map-Based Cloning of *essp5*

The mutant *essp5* was isolated from the same genetic screen as *essp1* and *essp3* [5], [27]. For genetic mapping of the *essp5* mutation, mutant plants were crossed with wild type plants of the L*er* ecotype. A total of 644 homozygous *essp5* mutants were collected from the F2 segregating population. Genomic DNA extracted from these seedlings was used for PCR-based mapping with simple sequence polymorphism markers, and the *essp5* locus was mapped to a ∼127 kb genomic interval on BACs F11A17, T1N15 and F9P7 on chromosome one (17,852–17,979 kb). Sequencing of the genomic region revealed a mutation in At1g48410.

### Histochemical GUS and Fat Red Staining

The modified GUS staining solution (0.5 mg/mL 5-bromo-4-chloro-3-indolyl-glucuronide, 20% methanol, 0.01 M Tris-HCl, pH 7.0) was used [5]. Seedlings immersed in GUS staining solution were placed under vacuum for 15 min, and then incubated at 37°C overnight. The staining solution was removed and samples were cleared by sequential incubation in 75% and 95% ethanol. Fat red staining was performed by incubating samples in a saturated solution of Sudan red 7B (Sigma) in 70% ethanol for 1 h at room temperature. Samples were then rinsed with 70% ethanol [57].

### Gene Expression Analysis by qRT–PCR and Northern Blotting

Plants grown on MS media were used for gene expression analyses. RT-PCR and RNA blot analyses were preformed as described previously [5]. Probes for detecting transcripts of the *CRA1*, *CRB*, and *CRC* genes were designed based on Pang et al [58]. Real-time PCR was conducted using the Bio-Rad CFX96 real-time PCR detection system and the SsoFast™ EvaGreen® Supermix kit (Bio-Rad Laboratories, Inc.). Data from three biological replicates were analyzed by the software Bio-Rad CFX96 Managertm V1.6.541.1028, using *Actin8* as the internal reference. DNA oligonucleotides used as probes or in real-time PCR are listed in Table S1.

### miRNA Northern Blot Analysis

RNA isolation and hybridization for miRNA detection were performed as described [59], [60]. 5′-end-labeled ^32^P antisense DNAs or an LNA oligonucleotide (for miRNA166) were used to detect miRNAs from total RNAs (10 µg each sample). Oligonucleotide probes used are listed in Table S1.

### miRNA Transgene Constructs

Genome sequences surrounding the selected *MIRNA* genes (listed in Table S1) were amplified by PCR from genomic DNA isolated from wild-type Arabidopsis (Col). The amplified DNA was first cloned into the pDNR221 vector (Invitrogen), confirmed by sequencing, and then recombined into the pEarlyGate100 Gateway-compatible destination vector [61] where the *MIRNA* genes are under the control of the CaMV 35S promoter. The constructs were introduced into *essp5* homozygous or heterozygous plants (*essp5/+*). PCR primers used for amplifying the *MIRNA* genes are listed in Table S1. Transgenic plants were selected on Basta, allowed to grow to maturity and seeds were collected, and GUS expression was analyzed in the next generation.

### ChIP

For the construction of the *PHB:PHB G202G-YFP* transgene plasmid, the *PHB* promoter was PCR amplified from Arabidopsis (Col-0) genomic DNA by Fusion DNA Polymerase (NEB, M0530) using primers EcoRI-PHBpr and PHBpr-NcoI, and inserted into the pBluscript SK vector. The plasmid was then fully digested by SpeI and partially digested by EcoRI. The full-length promoter fragment was purified and ligated with the pEARLEYGATE 104 vector [61] to generate the plasmid *pEG104-PHBpro*. The *PHB G202G* coding sequence was amplified from cDNAs made from *35S:PHB G202G* transgenic plants [43] with primers PHBf and PHBr, cloned into the pENTR-D-topo vector (invitrogen), and subsequently cloned into the destination vector pEARLEYGATE104 by LR reaction. The generated plasmid *pEG104-PHB G202G* were digested by NcoI and SpeI, and the *PHB G202G-YFP* fragment was recovered and ligated with *pEG104-PHBpro* to obtain the *pEG104-PHB:PHB G202G-YFP* plasmid. The *PHB:PHB-YFP* transgene plasmid was constructed using a similar strategy. Primers are listed in Table S1.

Chromatin immunoprecipitation (ChIP) was carried out as described previously [62]. One gram of twenty-day-old Arabidopsis plants grown on MS agar was used for each ChIP. The sonicated chromatin was immunoprecipitated with 5 µL of anti-GFP antibody (ab290, Abcam). Quantitative ChIP PCR was performed with three technical replicates, and results were presented as percentage of input. ChIP experiments were performed at least two times. See Table S1 for primer sequences used for ChIP-PCR and construction of the *PHB:PHB G202G-YFP* transgene.

## Supporting Information

Figure S1 Morphological phenotype of mature *essp5* plants. (A) Morphological comparison of the *essp5* mutant with wild type (*βCG:GUS*) at maturity. (B) A close-up view of a single branch of a typical *essp5* plant.
(TIF)

Figure S2 Genetic Mapping of *essp5*. (A) Fine genetic mapping with PCR-based markers located the *essp5* locus to the bottom of chromosome 1, on BAC clones F11A17, T1N15, and F11I4. The numbers of recombination events out of the total numbers of chromosomes examined (1,288) are indicated. (B) Structure of the *AGO1* gene and the location of the *essp5* mutation. Boxes and lines represent exons and introns, respectively. The colored boxes represent the conserved protein domains: green (PAZ), blue (MID), red (PIWI). A single mutation (C2212 to T2212) was found in the 13th exon of *AGO1* (At1g48410). This mutation potentially leads to the replacement of Leucine with Phenyalanine at amino acid 740 of the protein.
(TIF)

Figure S3 Phenotype of F1 plants from the cross of *ago1-25* and *essp5*. (A) Morphological comparison of *ago1-25*, *essp5* and the F1 progeny (*ago1-25 essp5*) at 45 days. (B–D) GUS phenotype of *ago1-25*, *essp5* and the F1 progeny (*ago1-25 essp5*) grown on MS agar for 14 days. Scale bars, 2 mm.
(TIF)

Figure S4 qRT-PCR analysis of *PHB* expression in wild type and miR166 overexpressors. Plants were grown for 14 days on MS agar. The miR166 overexpressors analyzed here were the wild type siblings of *miR166ox-*1 and *miR166ox-2* shown in Figure 5 (the miR166 construct was initially introduced into an *essp5* heterozygous background). *Actin-8* was used as an internal control. The mean and standard error were determined from two biological replicates.
(TIF)

Figure S5 ChIP analyses of PHB occupancy at seed maturation gene promoters. (A) Structures of the four master regulatory genes of seed maturation. Boxes and lines represent exons and introns, respectively. Transcription start sites are indicated by arrows. Black bars labeled P1–P3 represent the regions examined by ChIP-PCR and/or ChIP-qPCR. (B) PHB occupancy at the promoter regions of the four master regulatory genes of maturation by PCR analysis of the DNAs co-immunoprecipitated with GFP-specific antibodies (IP). Chromatin isolated before immunoprecipitation (input) served as a positive control. DNAs from a mock control (no antibody, no ab) and DNAs precipitated from a GFP only transgenic line (*35S:GFP*) served as negative controls. 1, no antibody; 2, *35S:GFP*; 3, *PHB:PHB G202G-YFP* (hemizygous, HE); 4, *PHB:PHB G202G-YFP* (homozygous, HO). PCR cycle numbers: 25 for input DNAs and 35 for ChIP DNAs. The results were reproducible in two independent experiments.
(TIF)

Figure S6 Transverse section of GUS-stained *essp5* leaf. The GUS stained leaf tissue was fixed using 2.5% glutaraldehyde in 4% paraformaldehyde and dehydrated through a graded series of ethanol. The sample was then embedded in LR White resin (Sigma-Aldrich) following the manufacturer's instructions. Serial 2 µm sections were cut by a Reichert-Jung Ultracut E Microtome equipped with a glass knife. The sections were mounted onto glass slides and observed under a Zeiss Axioskop 2 Plus microscope. Sacle bar, 20 µm.
(TIF)

Figure S7 PHB occupancy at *LEC2* gene promoter in developing siliques. (A) PHB occupancy at the *LEC2* promoter (P1 region as shown in Figure 7P) as determined by ChIP using anti-GFP antibody in siliques collected from *PHB:PHB-YFP* plants at 5-day after pollination. The *35S:GFP* plants served as negative control. ChIP DNAs were analyzed by qPCR. The results were reproduced in two biological replicates. Standard deviations were calculated from three technical repeats. (B) qRT-PCR analysis of *LEC2* expression in wild type and the *phb-13* mutant. Plants were grown for 14 days on MS agar. *Actin-8* was used as an internal control. The mean and standard error were determined from two biological replicates.
(TIF)

Table S1 Oligonucleotides used in this study.
(DOC)
