# Supplementary figures and images for: MicroRNA–Mediated Repression of the Seed Maturation Program during Vegetative Development in Arabidopsis
Source: PLoS Genet. 2012 Nov 29;8(11):e1003091. doi: 10.1371/journal.pgen.1003091 (PMC3510056; doi:10.1371/journal.pgen.1003091)

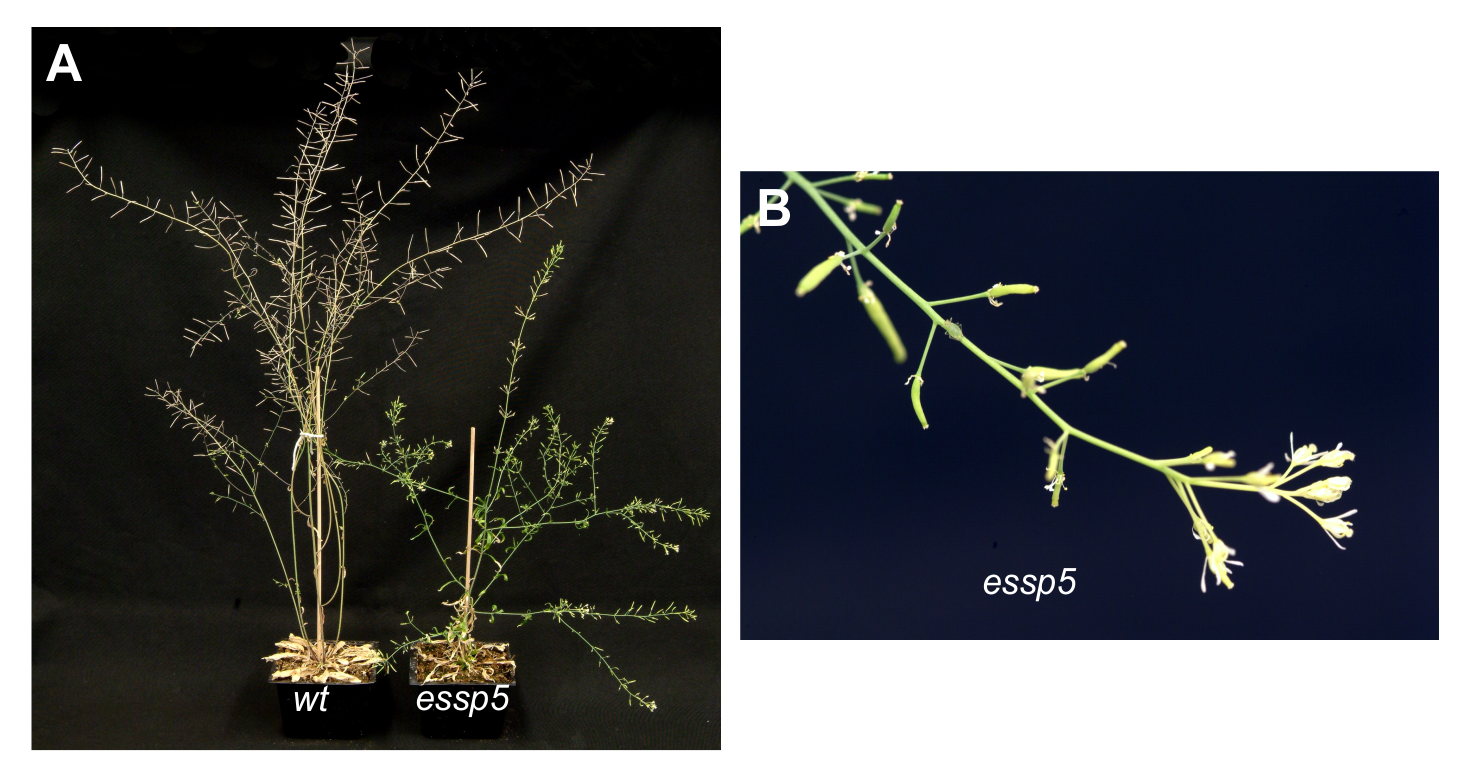

Supplement: Figure S1 — Morphological phenotype of mature essp5 plants. (A) Morphological comparison of the essp5 mutant with wild type (βCG:GUS) at maturity. (B) A close-up view of a single branch of a typical essp5 plant. (TIF) [file pgen.1003091.s001.tif]

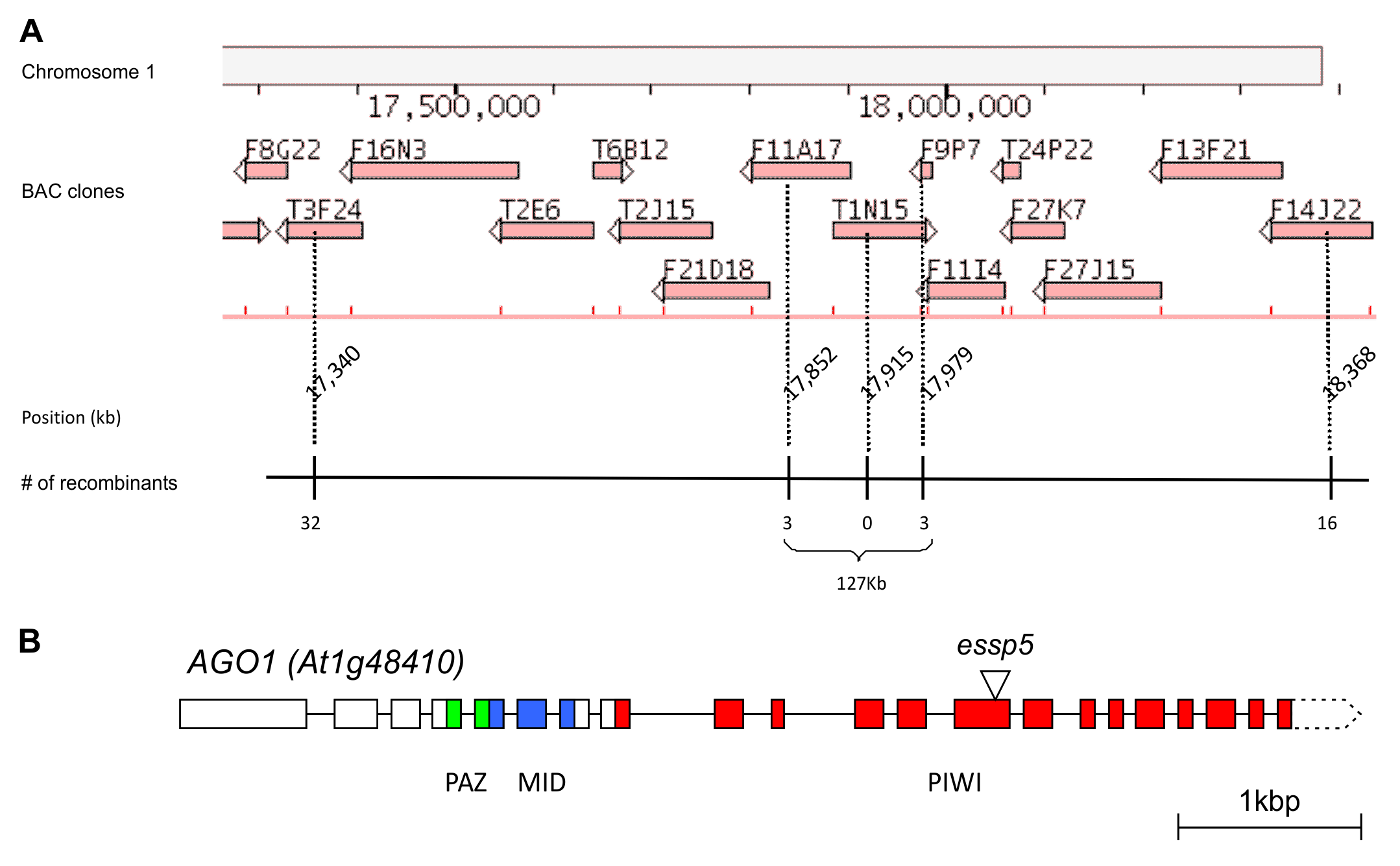

Supplement: Figure S2 — Genetic Mapping of essp5. (A) Fine genetic mapping with PCR-based markers located the essp5 locus to the bottom of chromosome 1, on BAC clones F11A17, T1N15, and F11I4. The numbers of recombination events out of the total numbers of chromosomes examined (1,288) are indicated. (B) Structure of the AGO1 gene and the location of the essp5 mutation. Boxes and lines represent exons and introns, respectively. The colored boxes represent the conserved protein domains: green (PAZ), blue (MID), red (PIWI). A single mutation (C2212 to T2212) was found in the 13th exon of AGO1 (At1g48410). This mutation potentially leads to the replacement of Leucine with Phenyalanine at amino acid 740 of the protein. (TIF) [file pgen.1003091.s002.tif]

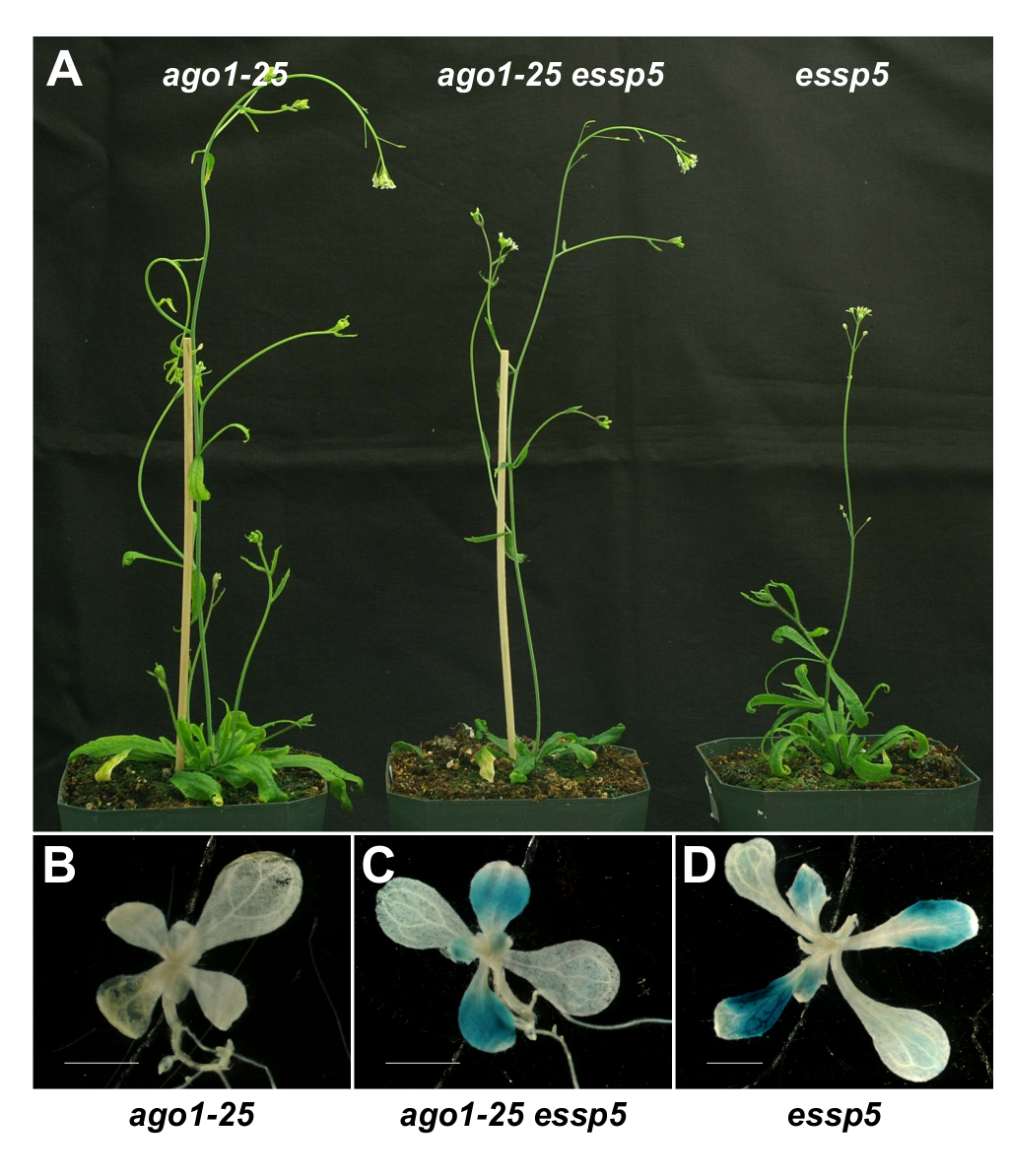

Supplement: Figure S3 — Phenotype of F1 plants from the cross of ago1-25 and essp5. (A) Morphological comparison of ago1-25, essp5 and the F1 progeny (ago1-25 essp5) at 45 days. (B–D) GUS phenotype of ago1-25, essp5 and the F1 progeny (ago1-25 essp5) grown on MS agar for 14 days. Scale bars, 2 mm. (TIF) [file pgen.1003091.s003.tif]

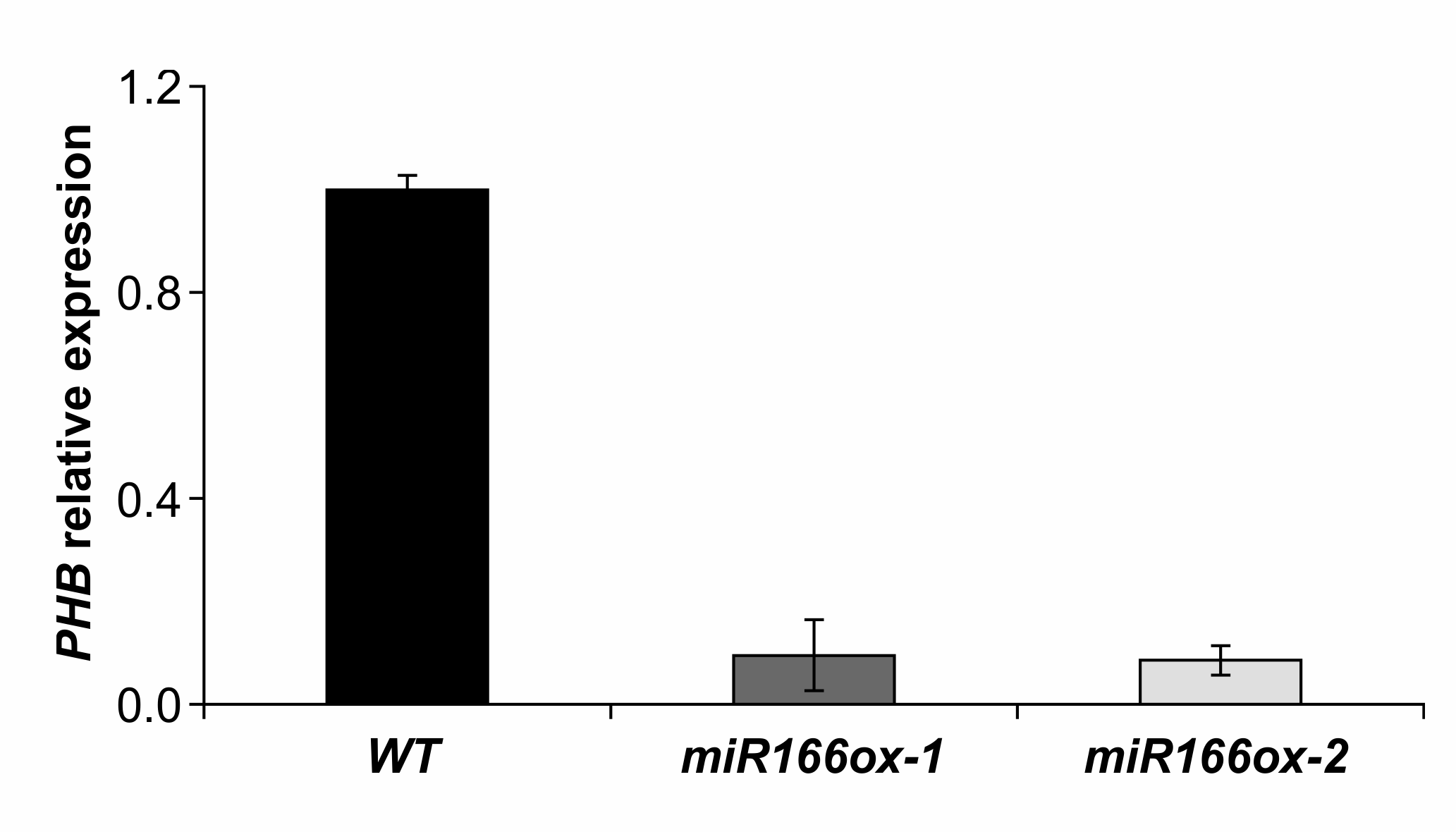

Supplement: Figure S4 — qRT-PCR analysis of PHB expression in wild type and miR166 overexpressors. Plants were grown for 14 days on MS agar. The miR166 overexpressors analyzed here were the wild type siblings of miR166ox-1 and miR166ox-2 shown in Figure 5 (the miR166 construct was initially introduced into an essp5 heterozygous background). Actin-8 was used as an internal control. The mean and standard error were determined from two biological replicates. (TIF) [file pgen.1003091.s004.tif]

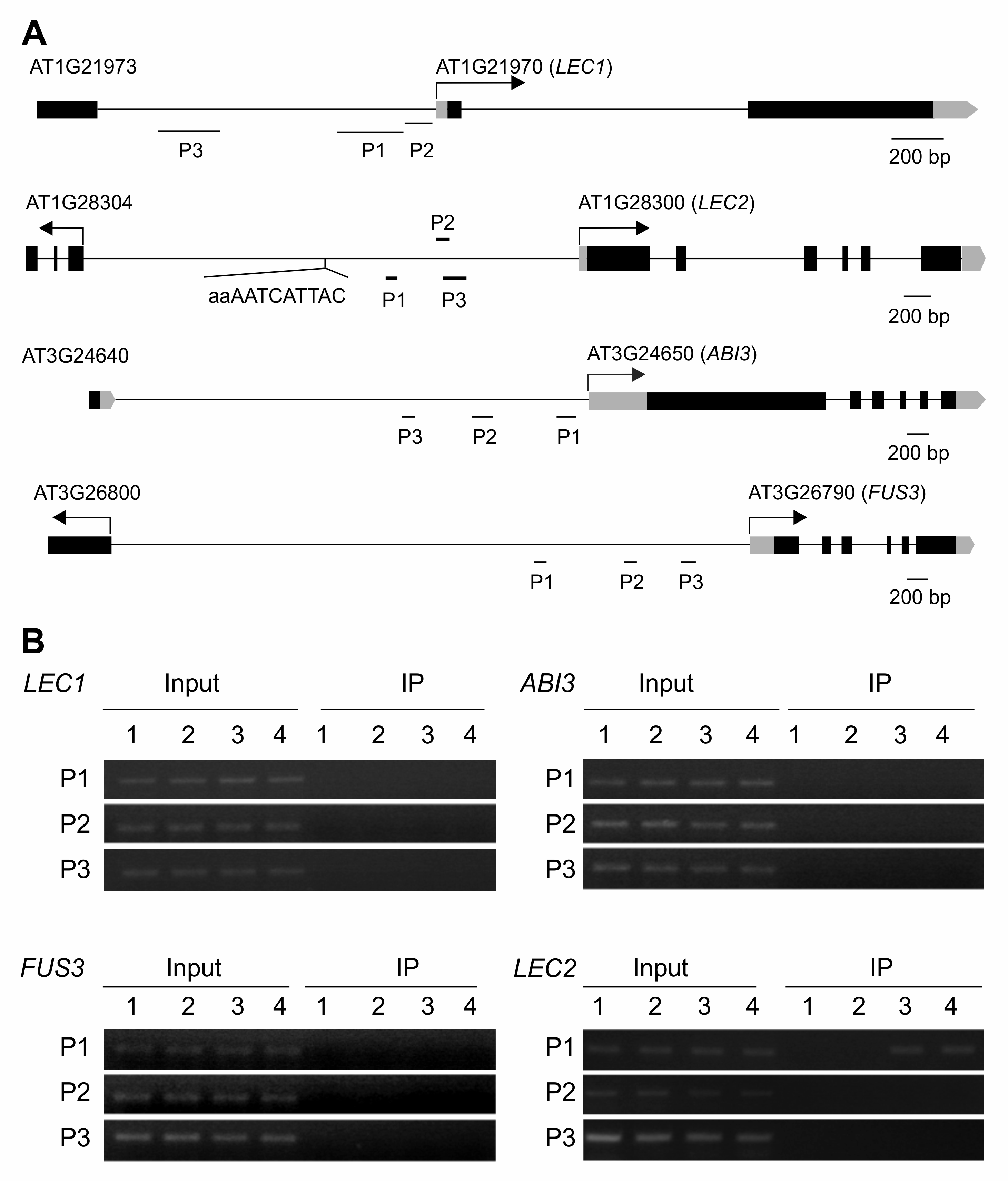

Supplement: Figure S5 — ChIP analyses of PHB occupancy at seed maturation gene promoters. (A) Structures of the four master regulatory genes of seed maturation. Boxes and lines represent exons and introns, respectively. Transcription start sites are indicated by arrows. Black bars labeled P1–P3 represent the regions examined by ChIP-PCR and/or ChIP-qPCR. (B) PHB occupancy at the promoter regions of the four master regulatory genes of maturation by PCR analysis of the DNAs co-immunoprecipitated with GFP-specific antibodies (IP). Chromatin isolated before immunoprecipitation (input) served as a positive control. DNAs from a mock control (no antibody, no ab) and DNAs precipitated from a GFP only transgenic line (35S:GFP) served as negative controls. 1, no antibody; 2, 35S:GFP; 3, PHB:PHB G202G-YFP (hemizygous, HE); 4, PHB:PHB G202G-YFP (homozygous, HO). PCR cycle numbers: 25 for input DNAs and 35 for ChIP DNAs. The results were reproducible in two independent experiments. (TIF) [file pgen.1003091.s005.tif]

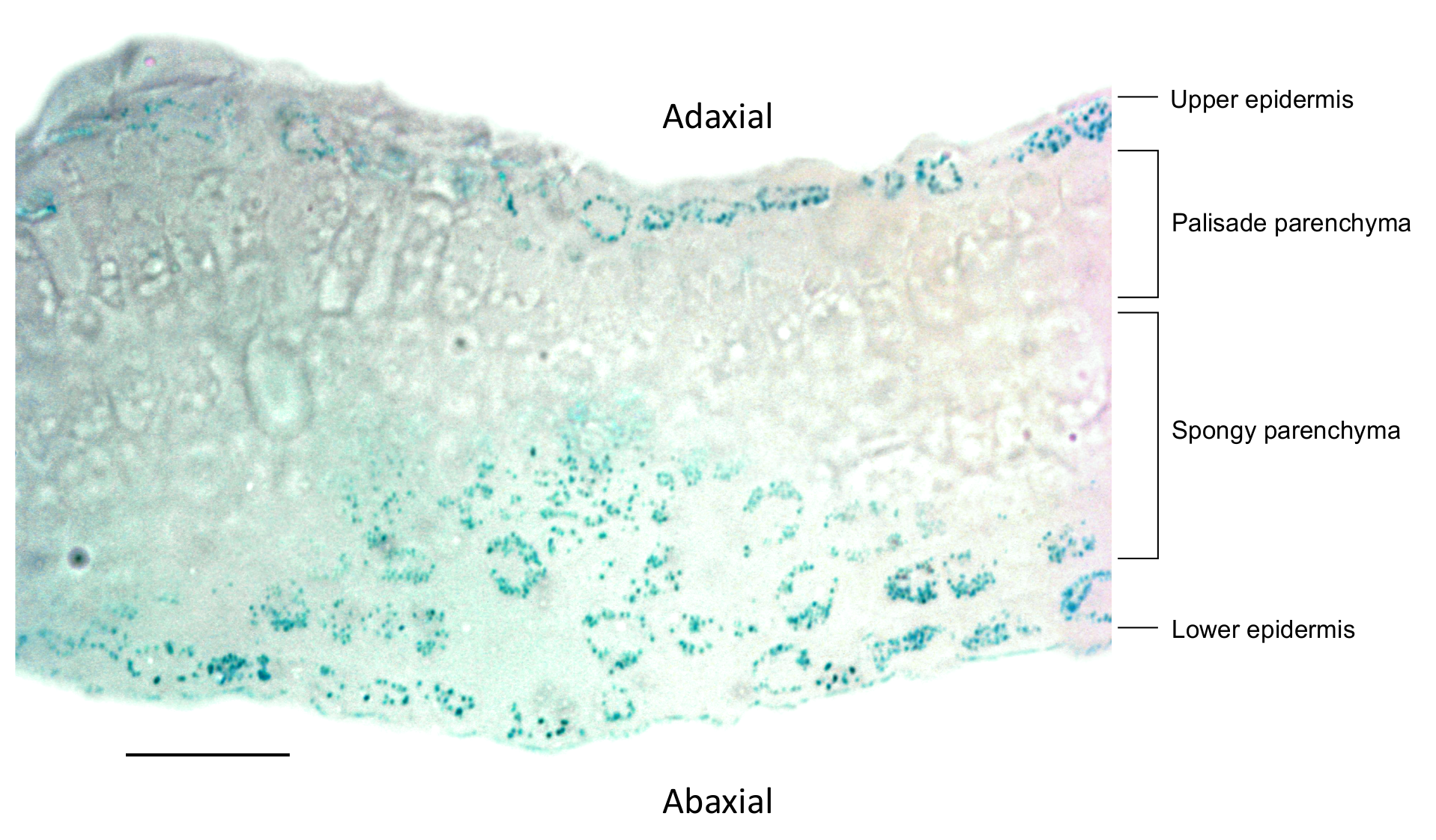

Supplement: Figure S6 — Transverse section of GUS-stained essp5 leaf. The GUS stained leaf tissue was fixed using 2.5% glutaraldehyde in 4% paraformaldehyde and dehydrated through a graded series of ethanol. The sample was then embedded in LR White resin (Sigma-Aldrich) following the manufacturer's instructions. Serial 2 µm sections were cut by a Reichert-Jung Ultracut E Microtome equipped with a glass knife. The sections were mounted onto glass slides and observed under a Zeiss Axioskop 2 Plus microscope. Sacle bar, 20 µm. (TIF) [file pgen.1003091.s006.tif]

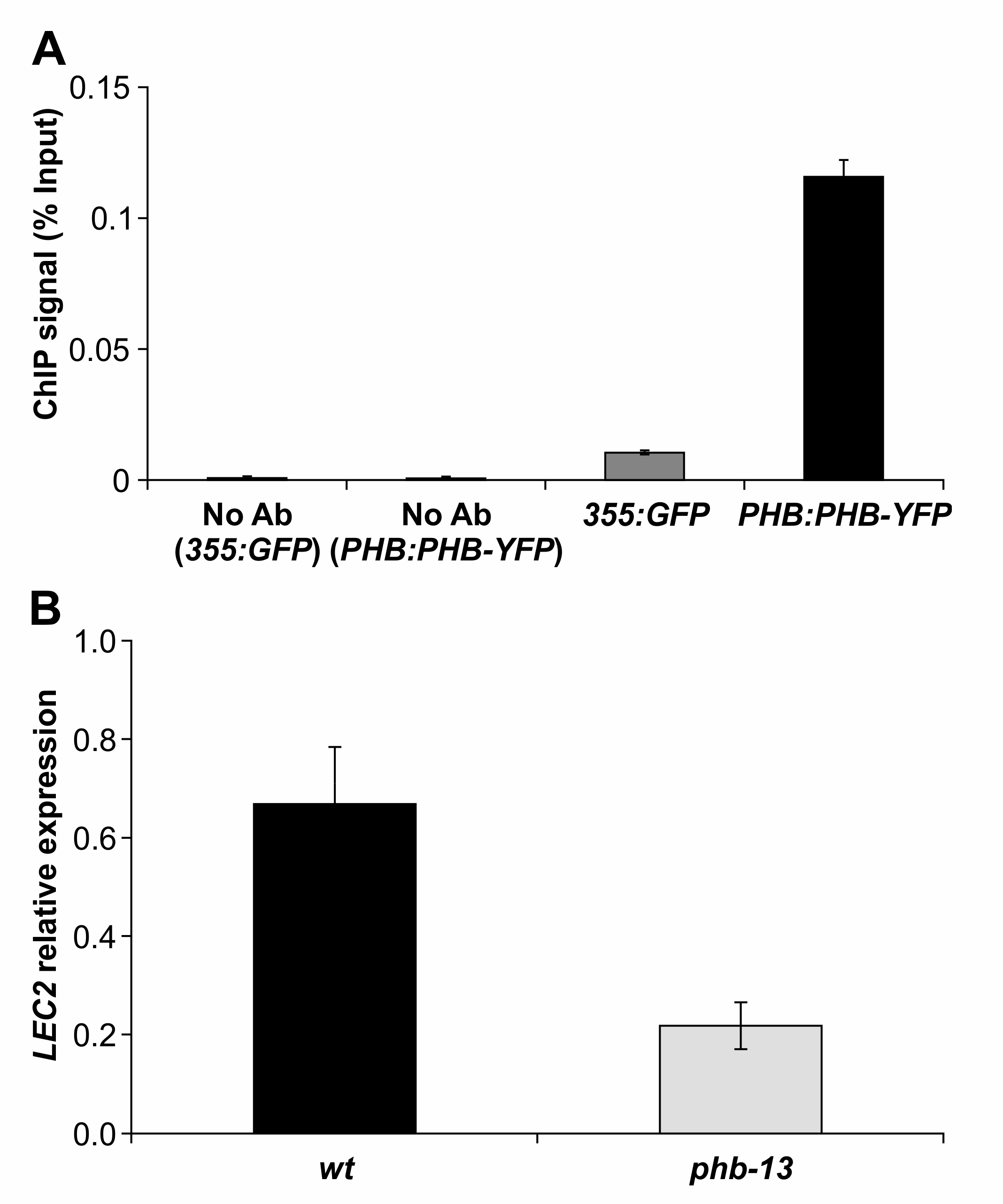

Supplement: Figure S7 — PHB occupancy at LEC2 gene promoter in developing siliques. (A) PHB occupancy at the LEC2 promoter (P1 region as shown in Figure 7P) as determined by ChIP using anti-GFP antibody in siliques collected from PHB:PHB-YFP plants at 5-day after pollination. The 35S:GFP plants served as negative control. ChIP DNAs were analyzed by qPCR. The results were reproduced in two biological replicates. Standard deviations were calculated from three technical repeats. (B) qRT-PCR analysis of LEC2 expression in wild type and the phb-13 mutant. Plants were grown for 14 days on MS agar. Actin-8 was used as an internal control. The mean and standard error were determined from two biological replicates. (TIF) [file pgen.1003091.s007.tif]
