# Supplementary material for: MicroRNA–Mediated Repression of the Seed Maturation Program during Vegetative Development in Arabidopsis
Source: PLoS Genet. 2012 Nov 29;8(11):e1003091. doi: 10.1371/journal.pgen.1003091 (PMC3510056; doi:10.1371/journal.pgen.1003091)
Supplement: Table S1 — Oligonucleotides used in this study. (DOC) [file pgen.1003091.s008.doc]

**Table S1. Oligonucleotides Used in This Study**

**Gene-specific primers pairs for qRT-PCR**

*LEC1* 5’-TGGACCAGCACAGCAACAACC-3’

5’-TTTGGCGTGAGACGGTAAGG-3’

*LEC2* 5’-GAAAGACCAAGACTGCATAGA-3’

5’-GATAGCTACCAACAGTCCAC-3’

*ABI3* 5’-TGGAACAGGACGATGATCTCGC-3’

5’-TTACCACCGCCTAGTCTTCTTGC-3’

*FUS3* 5’-AAGGATGCCTAGACAGAGACGATC-3’

5’-CGGGAGTATCATACGTCGGAGAG-3’

*PHB* 5’-CCTTCAGTCAAAGACTCTGTCGG-3’

5’-GCACAAAGCACGCCACTACC-3’

*ACT8* 5’-GCCGATGCTGATGACATTCA-3’

5’-CTCCAGCGAATCCAGCCTTA-3’

**Gene-specific primers used for RNA blot***

*2S1* 5'-GGAACAACAGCTATTCCAGCA-3'

5'-(T7*)-CACGATGAGGTGTGGTATGTG-3'

*2S2* 5'-GGAAGACGACATCGAGAACC-3'

5'-(T7*)-AAAAGACACGATGAGGTGTGG-3'

*2S3* 5'-ACCATTCCAATCCAGGAAAAT-3'

5'-(T7*)-GCTAACCGTACGCCATTACAG-3'

*2S4* 5'-GCAGAGACGACAGCTACTCCA-3'

5'-(T7*)-TTAGCCTCAAACATCCCAAAG-3'

*2S5* 5'-CCCTCTTCATCCTCCTAGCC-3'

5'-(T7*)-ATGTTGCACCTGTTGCTGTC-3'

*CRA1* 5'-TTACGTGACAGACGGGGAAG-3'

5'-(T7*)-GAGTGTTGATTTGCGCGTTA-3'

*CRB* 5'-TTGAGCGTTTCGTCATTGAG-3'

5'-(T7*)-ACCCTTGACTCTGACCCTGA-3

*CRC* 5'-AACCATGGCAAGGACGACAG-3'

5'-(T7*)-TCACATGTTCCACCTTCTGG-3'

*EF-1α* 5'-TGCTGTCCTTATCATTGACTCCACCAC-3'

5'-(T7*)-TTGGAGTACTTGGGGGTAGTGGCATC-3'

*RNA polymerase T7 promoter sequences, 5'-TAATACGACTCACTATAGGG-3' or

5'-TAATACGACTCACTATAGGA- 3', were added to the beginning of each reverse primer.

**List of microRNAs overexpressed in *essp5* background and primers used for amplifying and cloning of the miRNAs**

*miR156*  5’-GGGGACAAGTTTGTACAAAAAAGCAGGCTGGAGTAAGACACGTGTAGAAATC-3’

5’-GGGGACCACTTTGTACAAGAAAGCTGGGTAACTTCAGGGTGAAGCACATTAG-3’

*miR159a*  5’-GGGGACAAGTTTGTACAAAAAAGCAGGCTGAGCAGATCTCGATGGAAGTAGAGCTCCTTAAAGTTC-3’

5’-GGGGACCACTTTGTACAAGAAAGCTGGGTAGCCTGCAGAAGAAGATGTAGAGCTCCCTTCAATCC-3’

*miR160c*  5’-GGGGACAAGTTTGTACAAAAAAGCAGGCTGAACCTGAATCAAAGCACTGGACC-3’

5’-GGGGACCACTTTGTACAAGAAAGCTGGGTAACTTCATTTCCTCTTCCCAACAGTT-3’

*miR162a*  5’-GGGGACAAGTTTGTACAAAAAAGCAGGCTGGGGCGCTTATCAGTCAACTACACACT-3’

5’-GGGGACCACTTTGTACAAGAAAGCTGGGTACTGATGTGTTTCGTTTGATCCG-3’

*miR164*  5’-GGGGACAAGTTTGTACAAAAAAGCAGGCTGAGAAGGTGTGTGATGAGCAAG-3’

5’-GGGGACCACTTTGTACAAGAAAGCTGGGTATCACCAAGGTGGAGTGGTCATG-3’

*miR166a* 5’-GGGGACAAGTTTGTACAAAAAAGCAGGCTGGTTAATCTTAGCCGGGTCTCGTG-3’

5’-GGGGACCACTTTGTACAAGAAAGCTGGGTACCATGCAAACAATCAATAACGC-3’

*miR167a*  5’-GGGGACAAGTTTGTACAAAAAAGCAGGCTGGCACCCACTTTCGACCCTTAAACTCTCCA-3’

5’-GGGGACCACTTTGTACAAGAAAGCTGGGTATGAAGCTAGGAAAGAGGAGCTTTG-3’

*miR168a*  5’-GGGGACAAGTTTGTACAAAAAAGCAGGCTGGCTCTCTTCTCTTTCTTCATATCCC-3’

5’-GGGGACCACTTTGTACAAGAAAGCTGGGTAAACATTTGGGCAAACAAAAGGAGAC-3’

*miR169a*  5’-GGGGACAAGTTTGTACAAAAAAGCAGGCTAACCTTAGCTTGAGTTCTTGCGAT-3’

5’-GGGGACCACTTTGTACAAGAAAGCTGGGTACTTCTATATAAACCCAGAGCGGG-3’

*miR171a*  5’-GGGGACAAGTTTGTACAAAAAAGCAGGCTGGTCCATTTCCTTCTCTCTTACCCTAA-3’

5’-GGGGACCACTTTGTACAAGAAAGCTGGGTACCCTACAACAGATTTATACTTGGCA-3’

*miR172a*  5’-GGGGACAAGTTTGTACAAAAAAGCAGGCTGGAAAAATGGAAGACTAATTTCCGGAG-3’

5’-GGGGACCACTTTGTACAAGAAAGCTGGGTAAGCTTGTGGATCTATTAATGTCTTG-3’

*miR319a* 5’-GGGGACAAGTTTGTACAAAAAAGCAGGCTGGTGGAGTAGATGTACAAACACACGCT-3’

5’-GGGGACCACTTTGTACAAGAAAGCTGGGTATTCTTCACCTATCCATGGCGAT-3’

*miR390a*  5’-GGGGACAAGTTTGTACAAAAAAGCAGGCTGGTTAACGAAGAGGAGATGACGTGTG-3’

5’-GGGGACCACTTTGTACAAGAAAGCTGGGTATGCACCGGAGAAAGAGACTAAAG-3’

*miR393a*  5’-GGGGACAAGTTTGTACAAAAAAGCAGGCTGAGACGTCTGGTTTACTAGCTCCATAA-3’

5’-GGGGACCACTTTGTACAAGAAAGCTGGGTATTGAGATACAAGGTTGCGGAA-3’

*miR394a*  5’-GGGGACAAGTTTGTACAAAAAAGCAGGCTGAGCCAAGCTTATATAGCCCGTC-3’

5’-GGGGACCACTTTGTACAAGAAAGCTGGGTAACCCTAGATCGAGGCTCTTCA-3’

*miR403*  5’-GGGGACAAGTTTGTACAAAAAAGCAGGCTGGTGGGTTTAATTTGGCCCTTT-3’

5’-GGGGACCACTTTGTACAAGAAAGCTGGGTCAGAAATCATCAACGCAAGGG-3’

**Oligonucleotide probes for detecting microRNAs**

*miR159a* 5’-TAGAGCTCCCTTCAATCCAAA-3’

*miR163* 5’-ATCGAAGTTCCAAGTCCTCTTCAA-3’

*miR165* 5’-GGGGGATGAAGCCTGGTCCGA-3’

*miR166* 5’-GGGGAATGAAGCCTGGTCCGA-3’

*miR167* 5’-TAGATCATGCTGGCAGCTTCA-3’

*miR319a* 5’-GGGAGCTCCCTTCAGTCCAA-3’

*U6-I* 5’-GGCCATGCTAATCTTCTCTGTATCGTT-3’

*U6-II* 5’-CCAATTTTATCGGATGTCCCCGAAGGGAC-3’

**Primers for the *pPHB::PHB G202G-YFP* transgene construct**

*EcoRI-PHBpr*  5’-CGgaattcTCCACTCAATGTTATCTGTTCACA-3’

*PHBpr-NcoI*  5’-catgCCATGGagctcaaagtcagaaataggaaaa-3’

*PHBf*  5’-caccATGATGATGGTCCATTCGATG-3’

*PHBr*  5’-TCAAACGAACGACCAATTCAC-3’

**Primers used for ChIP-qPCR and ChIP-PCR analyses**

*LEC2-P1*  5’-TCGCCAAATGTAAGTTTCTGA-3’

5’-TCGTGCCCTAAGAACACCTC-3’

*LEC2-P2* 5’-TTTGCAAAATATGCACAAAATC-3’

5’-TGCGCTAAAATTTATTGGAATC-3’

*LEC2-P3*  5’-TATGCCGTGTGTTCTGATTCC-3’

5’-TCCAATATTGAAAAAGATAATTCCA-3’

*LEC1-P1*  5’-GGTCTATATCTCTTTCCCATTGGC-3’

5’-TTCTCAGCCGTTCGATAATCG-3’

*LEC1-P2* 5’-GATTTGTTGCATGCAAAGAGAGG-3’

5’-GCCAATGGGAAAGAGATATAGACC-3’

*LEC1-P3* 5’- ACCTGCATGTGGCCTCTTTAT -3’

5’- GAATCTTTCCTGGCCAATGAC -3’

*ABI3-P1* 5’-CTGCTGAGGTAATTGAATGCTGC-3’

5’-CCCATGTGTTCCAGTTTGTTCC-3’

*ABI3-P2* 5’-TTGAAGTGGAAGAGGCAGACAC-3’

5’-TTTGAAGGCAAGGTCGAACC-3’

*ABI3-P3* 5’-GGTGATGAATATGTGAGGTTGGG-3’

5’-CGTGTACGTTTAGGTGGCATG-3’

*FUS3-P1* 5’-GGACAAGTGGTATGCAATCCCTC-3’

5’-TTTGGAATGGTGTTGGATGGG-3’

*FUS3-P2* 5’-CGTCACGTATTCTTCCTCACCC-3’

5’-TTCTTGATAAGGGCGTTACTAAATG-3’

*FUS3-P3* 5’-CGGGCGTTACTAAATGAAAGTC-3’

5’-CTGATTTGGAAGAGAAGTGAAGG-3’
